# Transgenerational transmission of hedonic behaviors and metabolic phenotypes induced by maternal overnutrition

**DOI:** 10.1038/s41398-018-0243-2

**Published:** 2018-10-12

**Authors:** Gitalee Sarker, Rebecca Berrens, Judith von Arx, Pawel Pelczar, Wolf Reik, Christian Wolfrum, Daria Peleg-Raibstein

**Affiliations:** 1 Laboratory of Translational Nutrition Biology, Department of Health Sciences and Technology, ETH Zurich, 8603 Schwerzenbach, Switzerland; 2 The Babraham Institute, Babraham, Cambridge, CB223AT UK; 3 Center for Transgenic Models, University of Basel, Basel, Switzerland

## Abstract

Maternal overnutrition has been associated with increased susceptibility to develop obesity and neurological disorders later in life. Most epidemiological as well as experimental studies have focused on the metabolic consequences across generations following an early developmental nutritional insult. Recently, it has been shown that maternal high-fat diet (HFD) affects third-generation female body mass via the paternal lineage. We showed here that the offspring born to HFD ancestors displayed addictive-like behaviors as well as obesity and insulin resistance up to the third generation in the absence of any further exposure to HFD. These findings, implicate that the male germ line is a major player in transferring phenotypic traits. These behavioral and physiological alterations were paralleled by reduced striatal dopamine levels and increased dopamine 2 receptor density. Interestingly, by the third generation a clear gender segregation emerged, where females showed addictive-like behaviors while male HFD offspring showed an obesogenic phenotype. However, methylome profiling of F1 and F2 sperm revealed no significant difference between the offspring groups, suggesting that the sperm methylome might not be the major carrier for the transmission of the phenotypes observed in our mouse model. Together, our study for the first time demonstrates that maternal HFD insult causes sustained alterations of the mesolimbic dopaminergic system suggestive of a predisposition to develop obesity and addictive-like behaviors across multiple generations.

## Introduction

The global rapid rise in obesity, overweight and their associated comorbidities such as type 2 diabetes, cardiovascular diseases as well as mental health disorders over the past two decades become a major public health concern^1^. Therefore, it is of utmost importance to further understand the underlying mechanisms and to find ways for early prevention. The causes of obesity are multifactorial and may be a complex interaction of genetic and environmental factors^2^. Although genetic factors play an important role^3^, it cannot explain the rapid rise of obesity in the past decades^4^. The Barker hypothesis postulates that environmental insults during critical periods of development cause irreversible physiological changes later in life in the form of various diseases^5^. The increased prevalence of maternal obesity has raised new interest to study obesity in the light of this process in recent years^6^. Thus, epidemiological studies have revealed associations between maternal obesity and the increased susceptibility to develop obesity and the metabolic syndrome in the progeny later in life^7,8^. However, epidemiological studies are often unable to determine the specific link due to the presence of other confounding factors such as, parental socioeconomic conditions, postnatal life style factors, and genetic predisposition. Therefore, studies on experimental animals become essential to establish the causal links between maternal obesity and subsequent disease risk in subsequent generations. Experimental animal studies have demonstrated that maternal high fat-diet (HFD) exposure can alter the hypothalamic appetite regulatory system of the offspring, leading to hyperphagia later in life^9–11^. Recently, studies have shown that maternal HFD exposure can also program the mesolimbic reward system that influences offsprings’ food preference and may explain the increased risk for developing obesity and the metabolic syndrome^12–14^. Such programming effect of the central reward circuit might also lead to predisposition to develop substance abuse disorders in the offspring, although relatively little attention has been given so far to explore this association. We have shown that maternal HFD increases the susceptibility to overconsumption of palatable foods in the first generation as well as enhanced sensitivity to drugs of abuse^14^. These altered hedonic behaviors are associated with reduced striatal dopamine levels, suggesting that a hypodopaminergic state of the mesolimbic reward system may be responsible for the higher vulnerability to develop addictive-like behaviors and altered metabolic phenotypes in the offspring. DNA methylation, histone modifications and non-coding RNAs are the three known epigenetic marks that have been implicated in transgenerational inheritance of the modified traits. Whilst there is emerging evidence of maternal stress^15^, toxins^16^, and undernutrition^17^ induced inheritance of disease susceptibility across multiple generations and its association with altered epigenetic marks, there is not much known about the transgenerational effects of maternal obesity or overnutrition. Most studies in rodents have demonstrated that altered metabolic phenotypes induced by maternal HFD exposure can be transmitted up to the second generaion (F2)^18–20^ and recently Dunn et al. have reported increased body length as well as body weight in female mice is transmitted up to the third generation (F3)^21^. In order to establish a true transgenerational epigenetic inheritance, it is crucial to study the phenotypic changes up to the third (F3) generation^22–24^. Moreover, the underlying mechanisms and the role of epigenetic marks for such transgenerational effects still remain to be explored. Further, to date no studies have explored the transmission of addictive-like phenotypes across multiple generations following maternal HFD exposure. Therefore, in the present study, we aimed to investigate if altered hedonic behaviors and metabolic phenotypes induced by a maternal HFD insult are transmitted to the second and the third generations through the paternal lineage. Furthermore, we analyzed whether such an insult also affects the mesolimbic dopaminergic circuitry and whether epigenetic methylation marks could be identified that would promote such an inheritance.

## Materials and methods

### Animals

C57BL/6N mice (10 weeks old) were obtained from Charles River, Germany. Mice were acclimatized for 2 weeks in a temperature and humidity controlled facility under a reversed light-dark cycle (lights off: 7:00–19:00) with chow food (Kliba-Nafag 3430, Klibamühlen; major nutrients: 18.5% crude protein, dry matter 88%, crude fat 4.5%, 54.2% NFE) and water ad libitum. All mouse experiments described in this study were carried out in strict accordance with the recommendations in the Animal Welfare Ordinance (TSchV 455.1) of the Swiss Federal Food Safety and Veterinary Office. It was approved by the Zurich Cantonal Veterinary Office, Switzerland.

### Experimental design

**Fig. 1:**
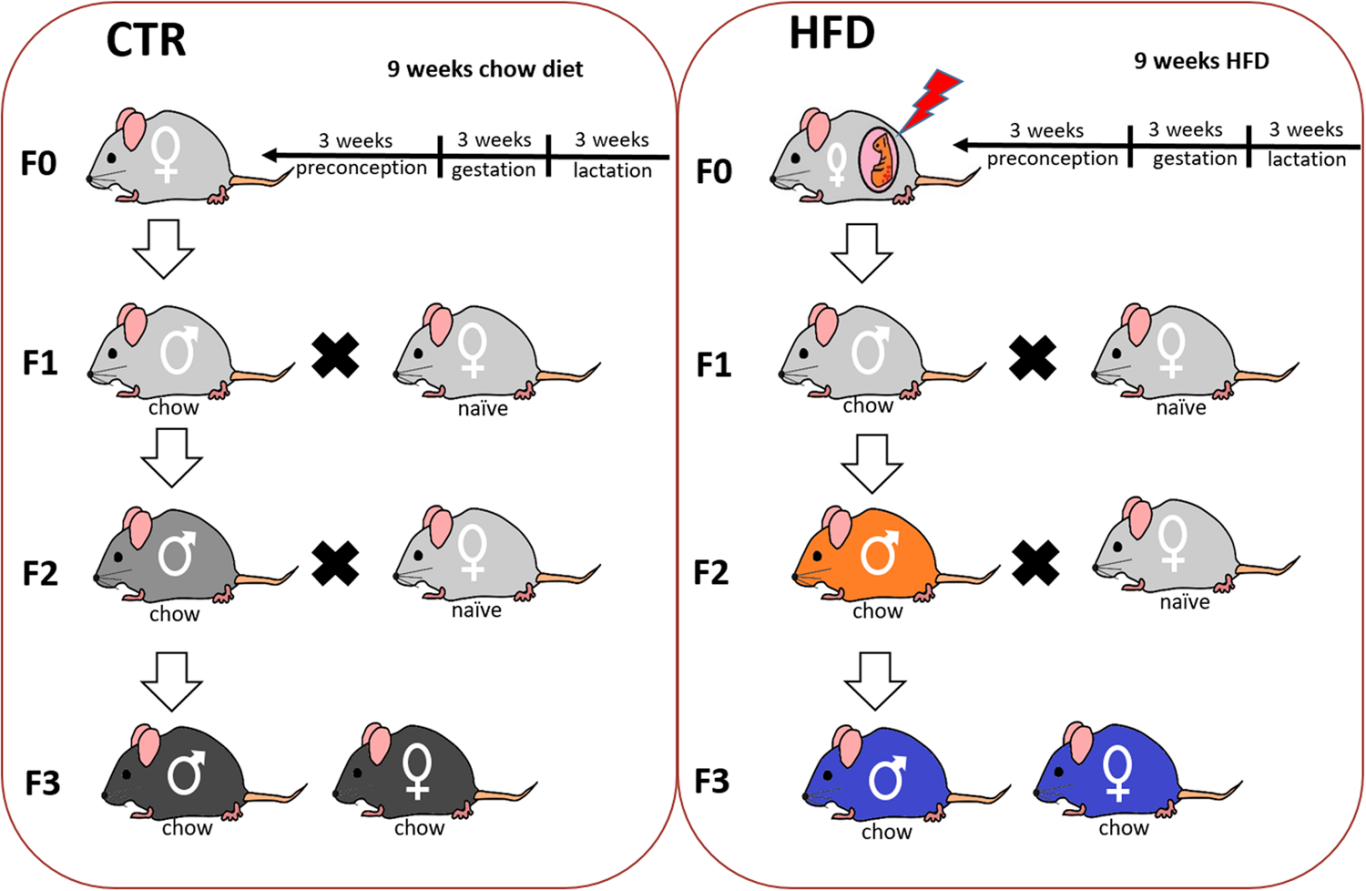
Breeding design used to generate F1, F2, and F3 progeny of HFD and chow fed dams. Female mice (F0) were fed either HFD or chow diet for 3 weeks prior to mating, 3 weeks during gestation and 3 weeks during lactation to obtain F1 HFD and F1 CTR offspring respectively. To generate F2 offspring through paternal lineage, F1 HFD and F1 CTR males were mated with naïve females. All pregnant dams were on chow diet throughout their pregnancy and lactation. F2 HFD and F2 CTR males were mated with naïve females to obtain F3 offspring. F1, F2, and F3 offspring from both groups were on chow diet since weaning. Colors of the mice are matched with the color codes used for the groups in subsequent graphs

To generate the F1 offspring, female mice were fed either a HFD (Kliba-Nafag 2127, Kaiseraugst, Switzerland; 60% energy from fat; major nutrients: 23.9% crude protein, dry matter 92%, crude fat 35%, 23.2% NFE) or normal laboratory chow (Kliba-Nafag 3430) for a total of 9 weeks: 3 weeks prior to mating, 3 weeks during gestation and 3 weeks during lactation^14,25^. Dams exposed to 9 weeks maternal HFD were not obese at the time of mating nor did they develop obesity or any altered metabolic phenotype as well as changes in maternal care during gestation and lactation^14^. Upon weaning on postnatal day (PND) 21 all the offspring were given ad libitum access to chow diet. To generate the F2 offspring, F1 males born to HFD exposed dams and chow fed dams were mated with naïve primiparous female mice reared on chow diet. The males were kept in the mating cage for 3–4 h until a vaginal copulation plug was confirmed. Pregnant dams from both groups were exposed exclusively to chow diet throughout gestation and lactation. Similarly, to generate the F3 offspring, F2 males from HFD and chow fed ancestors were mated with naïve primiparous females (Fig. 1). One offspring per litter from ten different dams was selected for hedonic, metabolic, neuroanatomical, and neurochemical tests to control for litter effect. Behaviorally naïve offspring were allocated for each experiment to avoid the possible effects of prior behavior testing. Both male and female offspring were included in order to assess potential sex-specific effects^26^. The groups were defined as HFD and control (CTR) in both F2 and F3 generations where offspring from HFD fed ancestors belong to the HFD group and offspring from chow fed ancestors belong to the CTR group. The experiments and evaluations commenced when offspring reached adulthood at PND 70.

In a separate cohort of animals, we have used in vitro fertilization (IVF) to generate F2 offspring by transferring F1 sperm into naïve females, in order to exclude the possible paternal exposure during mating. The behavioral phenotypes observed in HFD-IVF offspring were similar to those observed in F2-HFD offspring (Supplementary Fig. 9). This supports our hypothesis that male germ cells are the major direct mediator for the transgenerational inheritance of maternal HFD insults.

### Metabolic phenotyping

#### Body weight and fat composition

The body weight of the offspring was measured on a weekly basis from PND 28 till PND 91. Body fat composition was then assessed using a computed tomograpghy (CT) X-ray scanner. Detailed explanation of the procedure is included in the supplementary method section.

#### Insulin tolerance test

The test was conducted in 14-week old mice. Mice were fasted for 6 h. After measuring the baseline blood glucose levels, mice were given intraperitoneal (i.p.) injection of insulin (Actrapid; Novo Nordisk A/S) at a dose of 0.75 IU insulin/kg body weight in males and 0.6 IU insulin/kg body weight in females. Blood glucose levels were then monitored 15, 30, 60, 90, and 120 min after injection. Glucose level was measured in fresh tail blood using Accu-Chek Aviva device (Roche).

#### Metabolic cage study

The metabolic activity was assessed at the age of 20 weeks. O_2_ consumption, CO_2_ expiration, heat production, calorie consumption, locomotor activity, and respiratory exchange ratio (RER) were recorded during day and night using the TSE Phenomaster cages (TSE Systems GmbH, Bad Homburg, Germany). The description of the apparatus and the procedure is provided in the supplementary file.

#### Plasma parameters

Mice were fasted for 6 h prior to dissection. Blood was collected by means of cardiac puncture, mixed with 5 µl EDTA (0.6 M) and centrifuged on 1000 × *g* for 10 min to collect the plasma. Commercially available enzyme linked immunosorbent assay kit (ELISA; Crystal Chem Inc., USA) was used to measure plasma levels of insulin. Colorimetric assays were used to measure plasma levels of cholesterol (Chol; Roche, Switzerland), triacylglycerol (TG; Hitachi, Switzerland) and non-esterified free fatty acids (NEFA; Wako, Germany).

### Behavioral experiments

#### Fat preference

During the experiment, mice were caged individually in type II cages (14 × 18 × 34.5 cm) and had ad libitum access to water. Mice were presented with both laboratory chow diet and HFD for 3 h on 4 consecutive days. The consumption of laboratory chow and HFD as well as the animals’ body weight were measured after the test every day. HFD intake was calculated as an amount of consumed HFD in mg/g body weight per 3 h.

#### Sucrose and alcohol preference tests

The same protocol was employed for the sucrose and the alcohol preference tests. For the duration of the experiment, the mice were single housed in standard cages. Mice were first allowed to habituate to drink water from the two 15 ml polypropylene tubes for two days prior to the test. The preference tests started on the third day and were conducted for 9 days. The three different sucrose (0.5, 1, and 3%) and alcohol (2, 5, and 8%) concentrations were used. During the test, mice were given simultaneous free access to the two drinking tubes, one with water and one with test solution. The consumption of tested drinks was calculated as an amount of consumed solution in mg/g body weight per day.

#### Amphetamine-induced locomotor activity

The locomotor response to a systemic amphetamine administration (2.5 mg/kg body weight; intraperitoneal injection) was tested in an open field apparatus using the Ethovision system (Noldus Technology, Netherlands). The locomotor activity was indexed by the distance traveled in the entire arena, expressed as a function of 12 successive 10 min bins (See also supplementary file).

#### Conditioned place preference (CPP)

The CPP response to systemic cocaine administration at a dose of 20 mg/kg was assessed in the CPP chamber. The chamber consisted of two equally sized compartments with different colored walls and distinct floor textures that were separated by a small central area. At day 0 (pre-conditioning phase), mice were allowed to move freely in all chambers for 15 min. At days 1–8 (conditioning phase), half of the animals from each group received cocaine hydrochloride (Sigma–Aldrich, Switzerland) or saline (0.9% NaCl) in one compartment while the other half received it in the other compartment immediately before a 30 min placement in the assigned compartments. At day 10 (preference test), the mice were again given free access to the entire apparatus without any injection for 30 min. The distance traveled (successive 5 min bins) and total time spent (s) in each compartment were recorded by the Ethovision software. The detailed description of the procedure is provided in the supplementary file.

#### Behavioral sensitization to cocaine

The locomotor sensitization effect for cocaine was assessed in the CPP apparatus. Mice from both HFD and CTR groups received an intraperitoneal (i.p.) injection of cocaine at a dose of 20 mg/kg on days 2, 4, 6, 8 (during the conditioning phase of CPP test) and for the evaluation of behavioral sensitization, all the animals received a cocaine injection on day 21. The basal locomotor activity was measured for 30 min and cocaine-stimulated locomotor activity was measured for 60 min.

### Molecular experiments

#### Gene expression analysis of deltaFosB

On the last day of cocaine sensitization test, the mice were sacrificed 1 h post-cocaine injection and the different brain regions were extracted and stored at −80 °C. The expression of delta FosB in dSTR and Nac was then assessed by quantitative real time PCR (q-RT PCR). The detailed description of the methods used for brain extraction and q-RT PCR is in the supplementary method section.

#### Immunohistochemistry

The immunohistochemistry was performed in the adult behaviorally naïve mice using DAB immunostaining. The following primary antibodies were used: rabbit anti-tyrosine hydroxylase (TH) (Santa Cruz Biotechnology sc14007; diluted 1:500), rat anti dopamine transporter (DAT) (Chemicon MAB369; diluted 1:1000), rat anti-dopamine D1 receptor (D1R) (Sigma Aldrich D2944; diluted 1:1000) and rabbit anti-dopamine D2 receptor (D2R) (Chemicon AB5084P; diluted 1:500). The specificity of immunoreactions of the primary antibodies were assessed and validated in several recent publications^14,27,28^. Microscopic analysis and quantification were conducted using densitometry and steriological methods. The full description of the tissue processing for the immunohistochemistry and the image quantification methods are provided in the supplementary file.

#### Post-mortem HPLC

For post-mortem neurochemical analysis, the brain regions isolated from adult mice were processed usning 0.4 M perchloric acid at 4 °C. The levels of dopamine (DA) and its metabolites DOPAC and HVA were measured by a high performance liquid chromatography (HPLC) system as previously described^29^ (See also supplementary file for detailed description).

#### Preparation of sperm samples

Adult male HFD and control offspring from F1 and F2 generation were sacrificed by CO_2_ asphyxiation and sperm was collected as described previously^30^. The cauda epididymis and vas deferens were carefully removed and punctured to collect the sperm mass which was then suspended into 500 µl M2 medium (Sigma Aldrich, M7167). The sample was incubated at 37 °C for 1 h for capacitation. Following capacitation, the upper 2/3rd of the fluid containing sperm cells was collected in a fresh tube to eliminate somatic cell contamination. Sperm was pelleted by centrifugation at 10,000 rpm for 10 min and stored at −80 °C.

#### Sperm DNA extraction

Sperm DNA extraction was following Isolation of genomic DNA from sperm using the QIAamp® DNA Mini Kit.

#### Bisulfite conversion and sequencing analysis

Whole-genome bisulfite-sequencing libraries were generated using a post bisulfite-adapter tagging (PBAT) method as previously described^31^, using ten cycles of PCR amplification. Libraries were sequenced as single end libraries using Illumina HiSeq 2000. Six biological replicates of F1 each HFD and CTR group as well as seven biological replicates of F2 each HFD and CTR group were generated to ensure adequate power to detect statistically significant differences between the respective groups. CpG methylation calls were analyzed using R and SeqMonk software (http://www.bioinformatics.babraham.ac.uk/projects/seqmonk/). Graphing and statistics were performed using Seqmonk and RStudio. The anlysis is described in details in the supplementary file.

### Statistical analysis

Sample size was selected based on the common practice for behavioral experiments (*n* of 8–10) that ensures adequate power to determine a prespecified effect size and on our previous studies with the chosen methods^14^. Data were analyzed using the statistical software StatView (version 5.0). Analysis of variance (ANOVA) followed by post-hoc comparisons (Fisher’s least significant difference) or restricted ANOVA was employed whenever appropriate. For the weekly monitoring of body weight a 2 × 2 × 10 (maternal exposure × sex × weeks) repeated-measures ANOVA was employed. To analyze the preference for HFD a 2 × 2 × 2 (maternal exposure × sex × food) ANOVA was employed. For the sucrose and alcohol preference tests a 2 × 2 × 2 × 3 (maternal exposure × sex × preference substance × substance concentrations) repeated-measures ANOVA was used. The baseline and saline phases prior to the amphetamine injection were subjected to a 2 × 2 × 3 (maternal exposure × sex × 10-min bins) and amphetamine induced locomotor activity was subjected to a 2 × 2 × 12 (maternal exposure × sex × 10-min bins) repeated-measures ANOVA. In the test of CPP a 2 × 2 × 2 (maternal exposure × sex × drug-compartment) repeated-measures ANOVA was employed. Behavioral sensitization to cocaine was analyzed using a 2 × 2 × 6 (maternal exposure × sex × 10-min bins) repeated-measures ANOVA. For the postmortem neurochemical analysis each monoamine and its metabolites were subjected to a 2 × 2 × 6 (maternal exposure × sex × brain area) repeated-measures ANOVA. In the behavioral, metabolic, neuroanatomical and neurochemical tests, no difference was detecetd in the variance between the HFD and CTR groups (*F* > 1).

## Results

### Hedonic responses to natural rewards in the F2 and F3 offspring of HFD ancestors

We assessed altered hedonic responses to natural rewards (HFD and sucrose) in the F2 and F3 generations. In the HFD preference test, all offspring groups (both HFD and CTR) consumed more HFD than chow in both F2 (*F*_1,33_ = 117.552, *p* < 0.0001; Supplementary Fig. 1a) and F3 (*F*_1,36_ = 300.021, *p* < 0.0001; Supplementary Fig. 1b) generations, however no difference was detected in HFD preference between the groups. Similarly, in the sucrose preference test, both HFD and CTR offspring preferred the sucrose solution to water in F2 (*F*_1,35_ = 422.673, *p* < 0.0001) and F3 (*F*_1,32_ = 196.891, *p* < 0.0001) generations. Both groups (HFD and CTR) showed gradually increased sucrose consumption with higher concentrations of sucrose in the F2 (*F*_2,70_ = 163.739, *p* < 0.0001; Supplementary Fig. 1c) as well as in the F3 (*F*_2,72_ = 68.740, *p* < 0.0001; Supplementary Fig. 1d) generations. However, no difference in sucrose preference was detected between the groups.

### Addictive-like phenotype in the F2 and F3 offspring of HFD ancestors

**Fig. 2:**
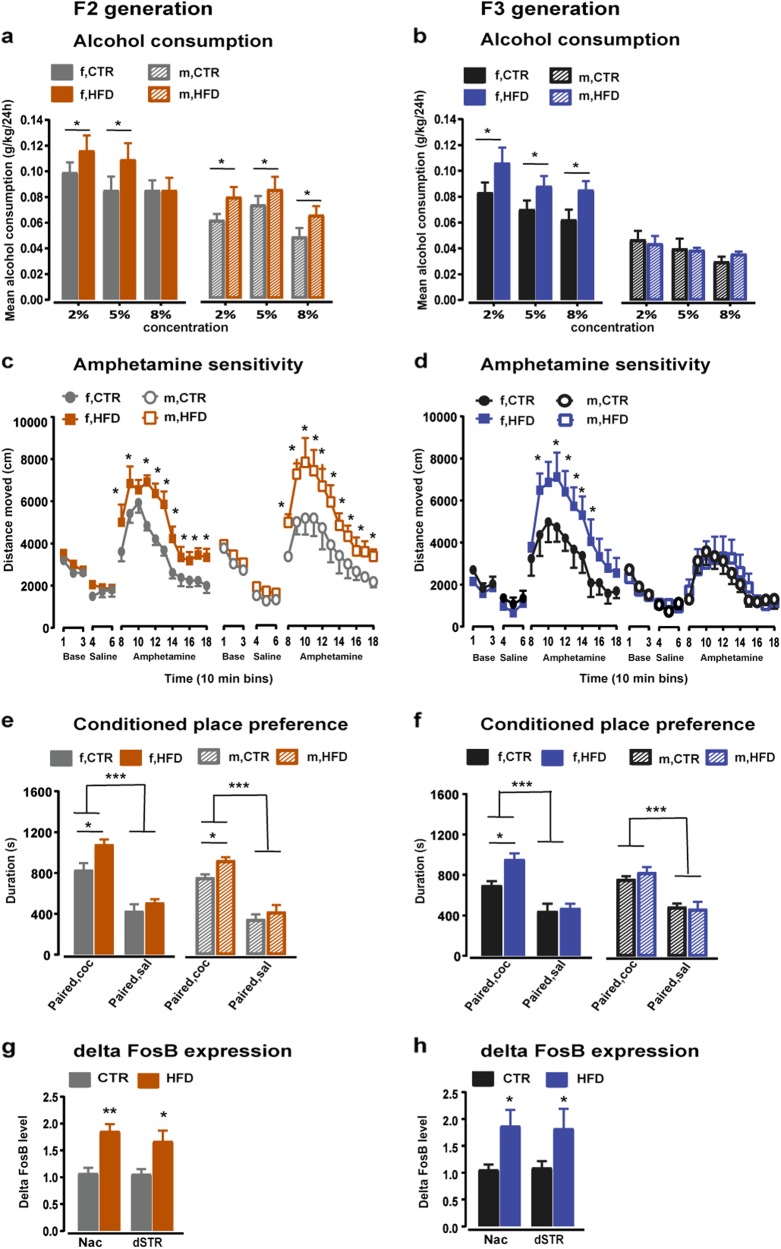
Addictive like phenotypes persistent in F2 and F3 offspring with HFD ancestors. **a**, **b** Alcohol preference in F2 and F3 generation respectively. The bar plots depict the mean alcohol consumption per day normalized to body weight in three different concentrations (2, 5, and 8%). *N* (F2 CTR) = 20 (10 m, 10 f); *N* (F2 HFD) = 20 (10 m, 10 f); *N* (F3 CTR) = 20 (10 m, 10 f); *N* (F3 HFD) = 20 (10 m, 10 f). **c**, **d** Represent the amphetamine induced locomotor activity in F2 and F3 offspring respectively. The line plots show the distance traveled in successive 10 min bins following baseline, saline and amphetamine injection in the open field. *N* (F2 CTR) = 12 (6 m, 6 f); *N* (F2 HFD) = 12 (6 m, 6 f); *N* (F3 CTR) = 12 (6 m, 6 f); *N* (F3 HFD) = 12 (6 m, 6 f). **e**, **f** Depict the conditioned place preference to cocaine in the F2 and F3 generation respectively. The bar plots show the total time spent in the cocaine-paired and saline-paired compartments. *N* (F2 CTR) = 24 (12 m, 12 f); N (F2 HFD) = 24 (12 m, 12 f); *N* (F3 CTR) = 24 (12 m, 12 f); *N* (F3 HFD) = 24 (12 m, 12 f). **g**, **h** Expression of delta FosB in the F2 and F3 offspring respectively. The bar plots represent the levels of delta FosB in the Nac and dSTR following cocaine sensitization. *N* (F2 CTR) = 12 (6 m, 6 f); *N* (F2 HFD) = 12 (6 m, 6 f); *N* (F3 CTR) = 12 (6 m, 6 f); *N* (F3 HFD) = 12 (6 m, 6 f). Data are represented as mean ± SEM. *p < 0.05, ***p < 0.0001. m, male; f, female

We further assessed the hedonic responses to alcohol and drugs of abuse in the F2 and F3 offspring. An alcohol preference test revealed that the F2-offspring preferred the alcohol solution to water (*F*_1,35_ = 71.886, *p* < 0.0001). However, both male and female HFD F2-offspring showed a significantly higher preference to alcohol compared to the controls (*F*_1,35_ = 7.098, *p* < 0.02; Fig. 2a). In the F3 generation, both offspring groups (HFD and CTR) preferred the alcohol solution to water (*F*_1,33_ = 9.625, *p* < 0.001), whilst female HFD F3-offspring consumed more alcohol in all three concentrations as compared to their CTR littermates (*p* < 0.01). Contrary, male HFD F3-offspring did not show any difference compared to the CTR offspring (Fig. 2b).

In an amphetamine sensitivity test, no difference in baseline locomotor activity and in response to a saline injection was detected between the F2-offspring groups. Thus, both male and female HFD F2-offspring showed enhanced locomotor activity in response to a systemic amphetamine injection compared to CTR F2-offspring (*F*_1,16_ = 13.258, *p* < 0.003; Fig. 2c). Similarly, the F3 generation did not differ in spontaneous locomotor activity levels and in response to the saline injection. However, amphetamine-induced locomotor activity was increased in female HFD F3-offspring compared to their controls (*p* *<* 0.001; Fig. 2d), whilst no difference was detected between male HFD F3-offspring and their controls.

In a CPP paradigm, while both F2-offspring groups (CTR and HFD) showed a higher preference to the cocaine- compared to the saline-paired compartment (*F*_1,40_ = 43.631, *p* < 0.0001; Fig. 2e); both male and female HFD F2-offspring spent more time in the cocaine-paired compartment compared to the CTR offspring (*F*_1,40_ = 4.773, *p* < 0.04; Fig. 2e). In the F3 generation, both groups displayed a preference to the cocaine- compared to the saline-paired compartment (*F*_1,40_ = 63.645, *p* < 0.0001; Fig. 2f). However, HFD F3-offspring showed a stronger preference to the cocaine-paired compartment compared to the CTR offspring (*F*_2,40_ = 7.185, *p* < 0.02; Fig. 2f). A subsequent post-hoc analysis revealed that female HFD F3-offspring showed a stronger preference to the cocaine-paired compartment as compared with their CTR littermates (*p* < 0.03).

The sensitization test was performed in the CPP paradigm following 21 days of withdrawal from cocaine. Whilst no difference in baseline activity levels was detected in the F2-offspring groups, both offspring groups showed enhanced activity levels in response to the challenge-cocaine injection (*F*_5,90_ = 10.310, *p* *<* 0.0001; Supplementary Fig. 2a). However, both female and male HFD F2-offspring displayed increased cocaine-induced locomotor activity as compared to their controls (*F*_1,18_ = 30.963, *p* < 0.0001). Similarly, both F3-offspring groups showed increased locomotor activity in response to the cocaine injection (*F*_5,95_ = 6.921, *p* < 0.0001; Supplementary Fig. 2b). However, HFD F3-offspring showed increased behavioral sensitization to cocaine as compared to CTR F3-offspring (*F*_1,19_ = 16.981, *p* < 0.001). Next, we determined the expression of delta FosB in the nucleus accumbens (Nac) and the dorsal striatum (dSTR) of F2 and F3 offspring following cocaine sensitization. HFD F2-offspring displayed higher levels of delta FosB in both the Nac and dSTR (*F*_1,20_ = 18.619, *p* < 0.001 and *F*_1,20_ = 6.380, *p* < 0.03, respectively) compared to the CTR offspring (Fig. 2g). Similar results of delta FosB levels were observed in HFD F3-offspring with increased levels in the Nac and dSTR (*F*_1,20_ = 11.795, *p* < 0.003 and *F*_1,20_ = 5.476, *p* < 0.03, respectively; Fig. 2h).

### Neuroanatomical alterations in the brain reward system in the F2 and F3 offspring born to HFD ancestors

**Fig. 3:**
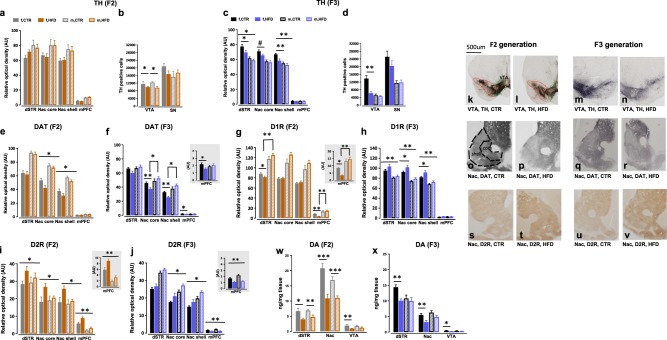
**Neuroanatomical and neurochemical alterations in the dopaminergic system of the F2 and F3- HFDoffspring**. Immunoreactivities of TH, DAT and D2R in dSTR and Nac (core and shell) were measured by densitometry and TH positive neurons were calculated in the VTA and SN by stereology. Levels of DA were measured in post-mortem brain tissue using high performance liquid chromatography (HPLC). **a**–**d** Expression of TH in the striatal regions, mPFC, VTA, and SN of the F2 and F3 offspring respectively. **e**, **f** Display DAT immunoreactivity in the dSTR, Nac (core and shell) and mPFC of F2 and F3 offspring respectively. **g**, **h** Represent the expression of D1R in the F2 and F3 offspring respectively. **i**, **j** Depict the levels of D2R in the dSTR, Nac (core and shell) and mPFC of F2 and F3 offspring respectively. **k**–**v** representative images of coronal brain sections of F2 and F3 offspring for the expression of TH in the VTA, DAT and D2R in Nac core and shell. **w**, **x** Display the levels of DA in the dSTR, Nac and VTA of F2 and F3 offspring, respectively. The inlet graphs in **f**, **g**, **i**, and **j** display a larger representation of the mPFC since DAT, D1R, and D2R expression are lower in this brain region. The content of monoamine is expressed as ng per mg fresh tissue weight. Data are represented as mean ± SEM. *p < 0.05, **p < 0.001, ***p < 0.0001. *N* (F2 CTR) = 16 (8 m, 8 f); *N* (F2 HFD) = 16 (8 m, 8 f); *N* (F3 CTR) = 18 (9 m, 9 f); *N* (F3 HFD) = 18 (9 m, 9 f) for immunohistochemistry. *N* (F2) = 20 (10 m, 10 f) per group; *N* (F3) = 18 (9 m, 9 f) per group for HPLC. TH, tyrosine hydroxylase, DAT, dopamine transporter, D2R, dopamine receptor 2, DA, dopamine, dSTR, dorsal striatum, Nac, nucleus accumbens, VTA, ventral tegmental area, SN, substantia nigra, mPFC, medial prefrontal cortex. m, male, f, female

To identify possible transgenerational effects of maternal HFD exposure on the central nervous system, we investigated and compared the expression of dopamine (DA)-related proteins, i.e., tyrosine hydroxylase (TH, the rate-limiting enzyme for dopamine synthesis), dopamine transporter (DAT) and dopamine receptor 1 and 2 (D1R and D2R) in the mesocorticolimbic reward system of F2 and F3 offspring. As shown in Fig. 3a, no group differences were observed in the F2 generation with respect to TH immunoreactivity in the dSTR, Nac-core and shell as well as medial prefrontal cortex (mPFC). In contrast, both male and female HFD F2-offspring displayed a reduction in the number of TH positive neurons in the VTA (*F*_1,18_ = 5.331, *p* < 0.03; Fig. 3b). In the F3 generation, HFD offspring displayed lower levels of TH in the dSTR (*F*_1,26_ = 5.963, *p* < 0.03; Fig. 3c), Nac-shell (*F*_1,26_ = 7.482, *p* < 0.02; Fig. 3c) and VTA (*F*_1,28_ = 9.930, *p* < 0.004; Fig. 3d). Subsequent post-hoc analysis yielded a significant reduction in TH immunoreactivity in the dSTR (*p* < 0.05), Nac-shell (*p* < 0.009) and VTA (*p* < 0.002) specifically in HFD female F3-offspring compared to the CTR female F3-offspring. DAT expression was lower in both male and female HFD F2-offspring in the Nac-core (*F*_1,28_ = 6.077, *p* < 0.03) and Nac-shell (*F*_1,28_ = 6.077, *p* < 0.02) compared to the CTR offspring (Fig. 3e). In contrast, a significant group × sex interaction was detected in DAT immunoreactivity in the Nac-core (*F*_1,27_ = 7.151, *p* < 0.02), Nac-shell (*F*_1,27_ = 8.883, *p* < 0.007) and mPFC (*F*_1,27_ = 4.85, *p* < 0.04) (Fig. 3f) in the HFD F3-offspring as compared to the CTR offspring. Subsequent post-hoc analysis revealed that female HFD F3-offspring showed lower expression of DAT in the Nac-core (*p* < 0.005), Nac-shell (*p* < 0.003) as well as mPFC (*p* < 0.02) compared to the CTR offspring. As shown in Fig. 3g, a significant interaction between group and sex was detected in the D1R immunoreactivity in the dSTR (*F*_1,29_ = 4.74, *p* < 0.04) and mPFC (*F*_1,29_ = 9.45, *p* < 0.005) in the HFD F2-offspring compared to CTR offspring. A subsequent post-hoc analysis revealed that HFD female F2-offspring displayed lower D1R expression in the dSTR (*p* < 0.05) and mPFC (*p* < 0.0001) compared to their controls. In contrast, HFD F3-offspring displayed increased expression of D1R in the dSTR (*F*_1,25_ = 4.455, *p* < 0.05), Nac-core (*F*_1,25_ = 9.572, *p* < 0.005) and shell (*F*_1,25_ = 9.15, *p* < 0.006) (Fig. 3h). Subsequent post-hoc analysis depicted that HFD female F3-offspring showed higher D1R immunoreactivity in the Nac-core (*p* < 0.04) and shell (*p* < 0.03). Furthermore, HFD F2-offspring displayed enhanced expression of D2 receptor in the dSTR (*F*_1,31_ = 4.209, *p* < 0.05), Nac-core (*F*_1,31_ = 5.438, *p* < 0.03), Nac-shell (*F*_1,31_ = 5.202, *p* < 0.03) and mPFC (*F*_1,31_ = 12.379, *p* < 0.002) (Fig. 3i). Similarly, HFD F3-offspring showed increased D2R immunoreactivity in the Nac-core (*F*_1,27_ = 9.325, *p* < 0.006), Nac-shell (*F*_1,27_ = 9.928, *p* < 0.005) and a tendency to higher D2R expression in the dSTR (*F*_1,28_ = 4.013, *p* = 0.05; Fig. 3j). In contrast, HFD F3-offspring displayed a significant reduction in D2R immunoreactivity in the mPFC (*F*_1,27_ = 23.966, *p* < 0.0001; Fig. 3j).

### Neurochemical alterations in the brain reward system in the F2 and F3 offspring

To support the immunohistochemistry findings, we measured the levels of DA and its metabolites 3,4 dihydroxyphenylacetic acid (DOPAC) and homovanillic acid (HVA) in VTA, Nac, dSTR, substantia nigra (SN), hypothalamus (Hypo), mPFC, amygdala (amyg), ventral (V. hip), and dorsal hippocampus (D. hip) of F2 and F3 offspring.

In the F2 generation, lower levels of DA were detected in the Nac, dSTR and VTA in HFD offspring as compared to their controls (*F*_8,230_ = 20.022, *p* < 0.0001; Fig. 3w). A subsequent post-hoc analysis yielded that both male and female HFD-offspring displayed reduced DA levels in the Nac (*p* < 0.0003) and dSTR (*p* < 0.03), whilst only female HFD offspring showed lower DA levels in the VTA (*p* < 0.03). In the F3 generation, decreased levels of DA were detected in the Nac, dSTR and VTA of HFD offspring (*F*_6,196_ = 2.349, *p* < 0.0326; Fig. 3x). A subsequent post-hoc analysis revealed that only female HFD F3-offspring showed lower DA levels in the Nac (*p* < 0.005), dSTR (*p* < 0.003), and VTA (*p* < 0.02) compared to their control littermates. The levels of DOPAC were reduced in the Nac (*p* < 0.04) of both male and female F2 HFD-offspring compared to their control littermates (Supplementary Fig. 4b). In the F3 generation, lower levels of DOPAC were detected in the VTA (*p* < 0.02) of HFD-female offspring (Supplementary Fig. 5b). As shown in Supplementary Fig. 4d, reduced HVA levels were detected in the Nac and the dSTR of HFD F2-offspring (*F*_8,230_ = 2.354, *p* < 0.02). A subsequent post-hoc test yielded that both male and female HFD-offspring displayed lower levels of HVA in the Nac (*p* < 0.0003), whilst only male F2-HFD offspring showed lower HVA levels in the dSTR (*p* < 0.04). In contrast, HFD female F3-offspring showed decreased HVA levels in the Nac (*p* < 0.007), dSTR (*p* < 0.003) and VTA (*p* < 0.001) (Supplementary Fig. 5d). No difference in the levels of DA, DOPAC, and HVA were detected in any other brain regions in both F2 (Supplementary Fig. 4a–c, and e) and F3 offspring (Supplementary Fig. 5a–c, and e).

### Obesity and insulin resistance development in second (F2) and third (F3) generation HFD offspring

**Fig. 4:**
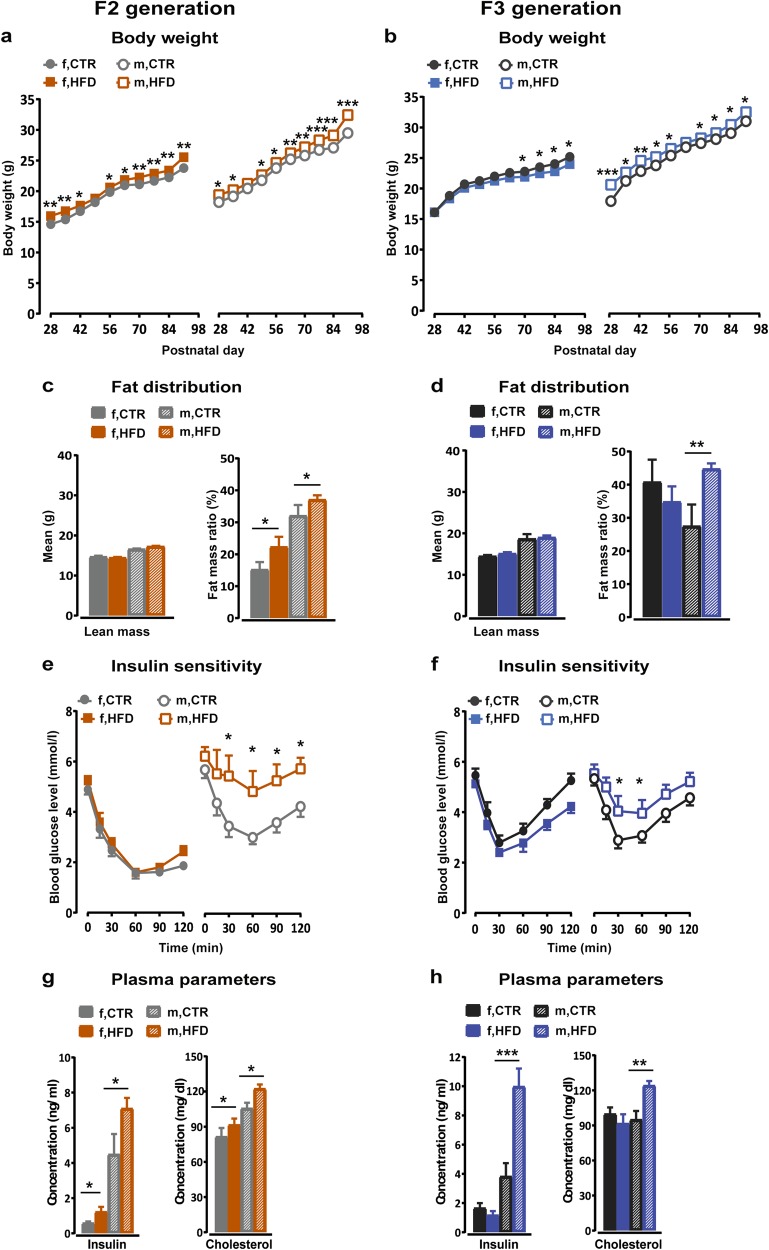
Altered metabolic phenotypes persistent in the F2 and F3 generation of HFD fed dams. **a**, **b** Body weight in the F2 and F3 generation respectively. The line plots depict the mean body weight per week for 11 consecutive weeks. *N* (F2 CTR) = 80 (48 m, 32 f); *N* (F2 HFD) = 86 (52 m, 34 f); *N* (F3 CTR) = 42 (22 m, 20 f); *N* (F3 HFD) = 52 (26 m, 26 f). **c**, **d** represent the distribution of fat in the F2 and F3 offspring, respectively. The bar plots show the average lean mass and percentage of fat mass ratio. *N* (F2) = 12 (6 m, 6 f) per group; *N* (F3) = 12 (6 m, 6 f) per group. **e**, **f** depict the insulin sensitivity in the F2 and F3 generation, respectively. The line plots show the blood glucose levels after 6 h fasting (time 0 min) and 15, 30, 60, 90, and 120 min following an insulin challenge injection. *N* (F2) = 16 (8 m, 8 f) per group; *N* (F3) = 16 (8 m, 8 f) per group. **g**, **h** show the metabolic profiles in the F2 and F3 offspring, respectively. The bar plots represent the concentrations of insulin and cholesterol. *N* (F2) = 12 (6 m, 6 f) per group; *N* (F3) = 12 (6 m, 6 f) per group. Data are represented as mean ± SEM. *p < 0.05, **p < 0.001, ***p < 0.0001. m, male; f, female

To identify possible metabolic transgenerational effects of perinatal maternal HFD exposure, we characterized the metabolic phenotype of F2 and F3 offspring. In the F2 generation, both male and female HFD offspring gained significantly more weight from PND 28 until PND 91 compared to the CTR offspring (*F*_9,144_ = 7.476, *p* < 0.0001; Fig. 4a). Further, F2-HFD offspring showed elevated body fat content (*F*_1,27_ = 4.83, *p* < 0.04) and higher fat mass in the visceral fat depot (*F*_1,27_ = 5.56, *p* < 0.03) (Supplementary Fig. 6a) as well as higher percentage of fat mass ratio (*F*_1,27_ = 5.009, *p* < 0.04) with no difference in lean body mass (Fig. 4c). An insulin tolerance test demonstrated significantly higher blood glucose levels in the HFD F2-offspring compared to the CTR F2-offspring after an insulin challenge injection (*F*_1,27_ = 6.702, *p* < 0.02; Fig. 4e). A subsequent post-hoc test supported a stronger insulin sensitivity impairment in HFD males F2-offspring (*p* < 0.05) compared to their controls. A lower metabolic rate was observed in both the dark and light cycle in HFD F2-offspring with reduced O_2_ consumption and CO_2_ production (*F*_1,20_ = 7.996, *p* < 0.02 and *F*_1,20_ = 8.699, *p* < 0.01, respectively; Supplementary Fig. 7a–b) compared with the CTR F2-offspring. In contrast, no difference was detected between the F2-offspring groups with regard to respiratory exchange ratio (RER), general locomotor activity and food intake (Supplementary Fig. 7d–f). Consistent with these findings, F2-HFD offspring also displayed significantly higher fasted plasma levels of insulin (*F*_1,20_ = 8.054, *p* < 0.02) and cholesterol (*F*_1,28_ = 5.911, *p* < 0.03) (Fig. 4g) as well as lower free fatty acids (FFA) (*F*_1,28_ = 15.163, *p* < 0.001; Supplementary Fig. 6e) compared to CTR offspring.

In the F3 generation, alteration in body weight was observed in HFD F3-offspring (*F*_1,85_ = 10.060, *p* < 0.003; Fig. 4b). A post-hoc test revealed that male HFD F3-offspring showed a significant increased body weight (*p* < 0.02) compared with the CTR male offspring, whilst female HFD F3-offspring weighed significantly less than the CTR female offspring from PND 70 (*p* < 0.05). The CT-scan of body composition revealed that male HFD F3-offspring showed increased total body fat content (*p* < 0.02), higher fat mass in both subcutaneous (*p* < 0.02) and visceral fat depot (*p* < 0.03) (Supplementary Fig. 6b) and higher percentage of fat mass ratio (*p* < 0.03) with no difference in the lean mass compared to the CTR male offspring (Fig. 4d). In addition, male HFD F3-offspring showed impaired sensitivity to insulin compared with the CTR offspring (*p* < 0.05; Fig. 4f). Furthermore, lower energy expenditure was detected in male HFD F3-offspring with lower CO_2_ production (*p* < 0.02) and lower RER (*p* < 0.04) (Supplementary Fig. 8b and d, respectively) as compared to the CTR offspring. Aligned with these findings, male HFD F3-offspring showed higher levels of circulating insulin (*p* < 0.01), cholesterol (*p* < 0.005) (Fig. 4h) and lower FFA levels (*p* < 0.008; Supplementary Fig. 6f) compared to CTR offspring. In contrast, female HFD F3-offspring showed lower levels of triglyceride (*p* < 0.02; Supplementary Fig. 6f) and no difference in any other plasma parameters when compared to CTR offspring.

### Analysis of CpG methylation in F1 and F2 sperm

**Fig. 5:**
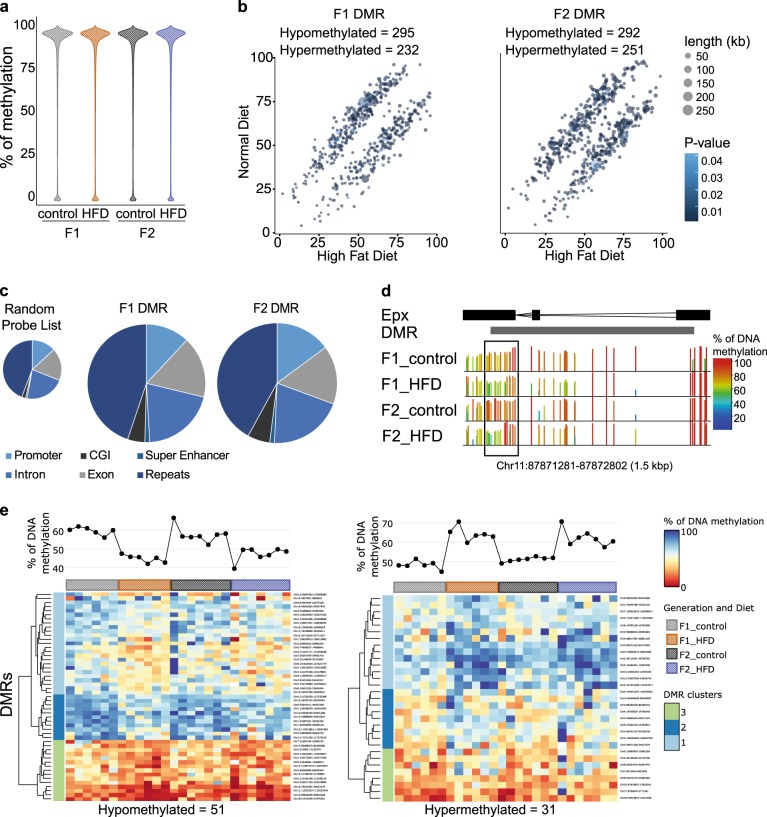
Analysis of sperm CpG methylation changes in F1 and F2 offspring. **a** Violin plots showing the distribution of CpG methylation levels measured by whole genome bisulfite sequencing (WGBS-seq) of sperm of F1 CTR (*n* = 6), F1 HFD (*n* = 6), F2 CTR (*n* = 7) and F2 HFD (*n* = 7) offspring. Methylated cytosines were counted for each rolling 50 CpG window genome-wide and are expressed as percentage of total cytosines per window. **b** (left) Comparisons of the percentage of sperm CpG methylation of differentially methylated regions (DMRs) found in F1 offspring. (right) Comparisons of the percentage of CpG methylation of DMRs found in F2 offspring. The size of the dot represents the size of the DMR and the blue color scale indicates the *p*-value of the DMR. **c** Genomic percentage of F1 and F2 DMRs in Promoters (light blue), CpG-islands (CGI) (dark gray), Super enhancers (dark turquoise), Introns (middle blue), Exons (light gray) and Transposons (dark blue). **d** Example DMR in the Epx gene. The size of the bar and the color indicates the methylation level, with high methylation level being red and low methylation level blue. **e** Heatmap of CpG methylation of significant DMRs in F1 which have a similar methylation patterns in F2. (left) hypomethylated DMRs in HFD, (right) hypermethylated DMRs in HFD. DMRs are clustered into 3 clusters unsupervised by k means clustering. The column mean % of methylation is shown as a graph above each heatmap. Heatmaps were generated using the iheatmap R library

Given the fact that we observed transgenerational inheritance of multiple traits into F3 which cannot be due to environmental exposures, we performed methylation studies to gain insights into epigenetic transgenerational inheritance by DNA methylation. In that respect our system is unique, as we are able to correlate the transmission of alterations in methylation marks with a very strong phenotype of certain traits over three generations. Therefore, we carried out whole-genome bisulfite sequencing (WGBS) of F1 and F2 offspring sperm. The global CpG methylation levels of the F1 and F2 sperm was 95% both in CTR or HFD offspring (Fig. 5a), consistent with previous measurements. To identify differentially methylated regions (DMRs) in F1 and F2 CTR in comparison to HFD, we defined DMRs as having a length of at least 100 bp and a methylation difference of at least 10% between CTR and HFD. Using this cutoff, we identified 527 DMRs in the F1 sperm (295 hypomethylated and 232 hypermethylated) and 543 DMRs in F2 sperm (292 hypomethylated and 251 hypermethylated) (Fig. 5b). Interestingly, even though we observe a strong penetrance of the metabolic/hedonic phenotype into the F3 generation we found only an overlap of 6 DMRs across the F1 and F2 generations, 3 being hypomethylated and 3 being hypermethylated (Supplementary Fig. 10) independent of a bias in the genomic distribution of DMRs among unique and repetitive elements (Fig. 5c). Although most significant DMRs in F1 offspring were not significantly differentially methylated in F2 offspring the same trend of differential methylation was still apparent in the F2 generation, with the methylation of the Epx gene serving as an example (Fig. 5d). Therefore, we analysed DMRs which were consistently hypomethylated or hypermethylated throughout the F1 and F2 generations, without the necessity of significance in the F2 generation. We found 82 DMRs which behaved consistently over both generations (51 hypomethylated and 31 hypermethylated) (Fig. 5e).

## Discussion

Our study demonstrates for the first time in a model of maternal HFD exposure which leads to addictive-like as well as an obesogenic phenotypes over three generations. These traits are transmitted by true transgenerational inheritance (i.e., into the third generation) in the absence of any further exposure to HFD. Notably, we observed a clear segregation of phenotypes between male and female HFD F3-offspring where female HFD offspring showed addictive-like phenotypes and male HFD offspring showed obesogenic phenotypes. For a clear overview, the main findings observed in F0 dams as well as F1, F2, and F3 offsprings are summarized in Table 1. The obesogenic and addictive like behaviors observed in HFD-F2 and F3-offspring were strongly supported by the sustained neuroanatomical and neurochemical alterations in the mesolimbic dopaminergic system. Methylome profiling of the sperm samples of F1 and F2 offspring revealed hundreds of significant differentially methylated regions (DMRs) between the HFD and CTR groups. While in a subgroup of these there was a pattern of conservation across the two generations, for only very few this reached statistical significance. Thus, it is possible that interactions between different methylation patterns occur across generations, which cannot be deconvoluted using the currently available methodology. Alternatively, it is possible that the sperm methylome might not constitute the carrier for the transgenerational transmission of the phenotypes observed in our model system.

**Table 1:** Summary of the observed behavioral and metabolic phenotypes across three generations

	F0 dams (HFD exposed; 3 weeks preconception, 3 weeks gestation and 3 weeks lactation)	F1 offspring		F2 offspring		F3 offspring	
		Male	Female	Male	Female	Male	Female
Hedonic response to drugs of abuse	NA	↑	↑	↑	↑	Ø	↑
Hedonic response to natural rewards	NA	↑	↑	Ø	Ø	Ø	Ø
Metabolic phenotypes	Ø	↑	↑	↑↑	↑↑	↑↑	Ø

In the table the summary of the observed behavioral and metabolic phenotypes of the F0 dams exposed to HFD and the subsequent F1, F2, and F3 offspring. Hedonic response to drugs of abuse referes to the alcohol preference test, amphetamine sensitivity test and the conditioned place preference response to cocaine. Hedonic response to natural rewards refers to the HFD and sucrose preference tests. Metabolic phenotypes comprise body weight (from weaning until adulthood), body fat assessment by CT-scan, insulin sensitivity and fasted plamsa insulin and lipid profiles. NA, not applicable; ɸ, no change; **↑↑**, increased; ↑, mild increase only detected from late adulthood (PND 120)

### Persistence of altered hedonic phenotypes across multiple generations

The present study is the first to show that maternal HFD exposure results in inherited addictive-like phenotype that persists across three generations. More specifically, HFD-F2 and HFD-female F3 offspring showed increased sensitivity and preference to drugs of abuse as well as a stronger cocaine-induced conditioned place preference response compared to the controls. Similar responses to drugs of abuse were observed in the HFD-F1 offspring^14^. In addition, F2 and F3-HFD offspring showed higher striatal delta FosB levels following cocaine sensitization. Delta FosB is well known for its role in long term neuronal adaption^32^ and overexpression of striatal delta FosB in rodents has been reported to be associated with enhanced sensitivity to cocaine and morphine^33,34^. The higher striatal delta FosB levels in our model may therefore indicate that HFD offspring are more sensitive to the incentive motivational properties of drugs of abuse.

The observed addictive-like phenotypes in HFD-F2 and F3 offspring are further supported by relevant alterations in the central reward system. Dopamine plays a crucial role in the mesocorticolimbic reward system and is involved in compulsive behaviors related to drug addiction and overconsumption of palatable foods that may lead to obesity^35^. Consistent with our findings in first generation HFD offspring, lower ventral tegmental TH levels in both F2 and F3-HFD offspring were detected. In addition, lower DAT expression and higher striatal D2 receptors density were observed in F2 and F3-HFD offspring. In accordance with these changes, lower DA levels in the VTA as well as in the striatum were observed. Similar alterations were reported in animal models of addiction following withdrawal from chronic ethanol^36^, cocaine^37^, and amphetamine^38^, which was associated with decreased VTA DA neuronal activity, lower striatal DA levels as well as altered D2R density. Such a hypodopaminergic state has been suggested to cause long lasting alterations in brain function that leads to drug-induced behavioral sensitization^39^. In human drug addicts, a hypoactive mesolimbic DA system is considered as potential trigger for compulsive drug intake and drug seeking behaviors^40^. A hypodopaminergic state has been linked to compulsive food intake in rodents where chronic HFD exposed mice showed increased body weight, which was correlated with higher D2R density and lower striatal DAT and DA levels^41^. Recently, it has been reported that insulin can interact with central reward system via modulating dopamine action^42^. In obesity and diabetic animal models, it was shown that insulin resistance leads to reduced DAT expression and lower DA levels in the Nac, resulting in increased palatable food intake^43^ and reduced sensitivity to drugs of abuse^44^. In addition, unpublished data from our lab showed that combined systemic injection of insulin and amphetamine in F1 HFD offspring reduced the amphetamine induced locomotor activity compared to control offspring. Taken together, our findings suggest that the hyporesponsive mesolimbic dopamine circuit and or insulin resistance might potentiate enhanced sensitivity and preference to drugs of abuse as well as leading to excessive weight gain in F2 and F3-HFD offspring. Therefore, it may suggest that subjects with a predisposition for obesity may share similar brain mechanisms to those with addictive-like behaviors.

In contrast, F2 and F3-HFD offspring do not show any enhanced preference to natural rewards such as sucrose and HFD compared to their control littermates. In a PET study, addicted individuals during withdrawal showed increased motivation when exposed to a drug stimuli and became less responsive when they were exposed to a food stimuli^45^. It has been suggested that addicted individuals develop a chronically elevated reward threshold and become unable to return to the baseline that makes them less responsive to natural rewards^40^. This might explain our finding where HFD F2 and F3-offspring show higher preference and sensitivity to drugs of abuse and not to natural rewards. Increased overconsumption of palatable foods might only be observed following exposure to a free-choice palatable diet over a long time period leading to insulin resistance and excessive weight gain, similar to the phenotype observed in the F1 offspring^14^. This pathological state can be explained by the decreased striatal insulin sensitivity which induces overconsumption of palatable food leading to obesity^41^.

### Persistence of obesogenic phenotypes across multiple generations

Different environmental exposures taking place early in life influence the phenotypic characteristics and increase the risk of diseases in the offspring. Recent studies which employed different early life insults, such as maternal overnutrition, maternal undernutrition, maternal infection, maternal deprivation and postnatal stress^15,17,20,21,46–51^ showed that besides genetic changes also epigenetic features may lead to heritable phenotypic alterations that are preserved through up to the second generation^15,17,20,52^. In recent years, several studies raised the importance of the epigenetic inheritance via the paternal lineage of early environmental insults^21,53–55^, since it is known that transmission via the maternal linage contains several confounding factors such as hormones, immune factors, influences from the oocyte, suboptimal uterine environment and maternal care^56–58^. However, in order to verify that a “real” transgenerational effect takes place, the non-exposed generation, namely F3 offspring, needs to be studied^21–24,46^. Here, we have shown that the metabolic phenotype via the paternal lineage was more pronounced in HFD F2- and HFD male F3-offspring with increased body weight, increased adiposity, resistance to insulin as well as significantly altered circulating metabolic parameters compared to the mild phenotypes observed in HFD F1-offspring^14^. A similar pattern of phenotypic inheritance in the second generation while skipping certain phenotypes in the first generation has been reported in an animal model of maternal cafeteria diet both via maternal and paternal lineages^18^. A possible explanation for such phenotypic inheritance in our model could be that maternal HFD exposure induces a mild effect on the F1 somatic cells related to metabolic phenotype and therefore, F1 offspring manifest moderate metabolic symptoms. Nevertheless, maternal HFD exposure is still able to reprogram the developing germ cells, that can transmit the altered phenotypic information to the subsequent generations and manifest stronger metabolic phenotypes in F2 and F3 offspring. In another study, contrary to our findings, only F3 female offspring born via the paternal lineage showed increased body length and increased body weight with normal insulin sensitivity, while F3 male offspring from the maternal and paternal lineages exhibit no differences in body length and body weight but showed enhanced glucose tolerance and normal insulin sensitivity^21^. Discrepancies in the findings between these two studies may be related to the timing and the duration of maternal exposure to the diet, dietary constituents (fat, protein, carbohydrate, and micronutrients), especially since opposite to our animal model Dunn and Bale reported that their maternal HFD exposure of 45% fat induced an obesogenic phenotype in the dams^20,21^.

### Sexual segregation in the inheritance of altered phenotypes in F3 generation

We observed a double dissociation of phenotypes in the third generation where HFD-male offspring showed an obesogenic phenotype and HFD-female offspring showed an addictive-like phenotype. At the molecular level, the hypodopaminergic state of mesolimbic reward system was more marked in the HFD F3-female offspring. Sex differences of phenotypic inheritance have been reported in a few recent studies of maternal HFD exposures^18,20,21,53^. However, the mechanism by which such segregation occurs still remains unclear. Differences in the maturation rates, the plasticity of the cellular events within female and male germ cells and the gonadal hormones have been reported as possible determinants of sex specific phenotypic inheritance^59,60^. Alternatively, the placenta may play an important role in sex specific fetal programming. Studies have reported an association between sex specific placental gene expression pattern and phenotypic inheritance in animal models of maternal stress^61^ and maternal diet^62^.

### Can epigenetic be considered as the underlying mechanism of transgenerational maternal HFD effects?

In order to further our understanding of the underlying mechanisms of transgenerational transmission of addictive-like and obesogenic phenotypes we analyzed the DNA methylation patterns in the sperm of F1 and F2 male offspring. We focused on sperm CG methylation, since it is one of the well characterized epigenetic marks and it is majorly implicated in transgenerational epigenetic inheritance^63^. Our findings are in line with other models of early-life nutritional insults such as maternal undernourishment^17^ and paternal overnutrition^54^ that have shown moderate changes (aprox. 10-20%) in cytosine methylation in sperm. Such moderate differences over a small number of CpGs are most likely to cause only penetrance of a phenotype across a set of siblings^64^. Further, environmental insults which induced alteration in epigenetic marks may depend on the onset, duration and the severity of the insults^63^. The latter environmental challenges are relatively severe compared to the perinatal HFD model employed in the present paper; indeed our model did not induce any overt metabolic effects in the mothers. Dams exposed to HFD (3 weeks preconception, 3 weeks during gestation and 3 weeks during lactation) did not show any alteration in body weight, fat mass ratio, blood glucose, insuiln and lipid profile compared to the chow fed dams^14^. Studies of nutritional insults exposure later in development only examined first generation offspring and were not associated with changes in sperm methylation^63,65^.

Together, our data suggest that sperm methylome might not constitute the major mode of transmission of the altered phenotypes to the subsequent generations following maternal HFD exposure. Given the strong metabolic and addictive-like phenotypes as well as alterations of the mesolimbic dopaminergic circuitry that were consistent until the third generation, we propose that other epigenetic carriers such as histone modifications and non-coding RNAs, may play a crucial role for such transmission. More specifically, small non-coding (snc) RNAs present in the male germ line have gained wide interest in recent years as an alternative mode of transgenerational epigenetic inheritance. Sperm sncRNAs regulate chromatin remodeling, DNA methylation, histone modifications and are important for germ cell development^66^. Recent studies have reported a mechanistic link between altered sperm sncRNAs and the transmission of metabolic and behavioral phenotypes in the progeny following parental stress^15^ or HFD exposure^55^. It will therefore be of great interest to examine in future studies the role of sperm sncRNAs in transgenerational transmission of altered metabolic and addictive-like phenotypes in transgenerational models of maternal HFD insult.

## Electronic supplementary material

Supplementary File

Supplementary Figure 1

Supplementary Figure 2

Supplementary Figure 3

Supplementary Figure 4

Supplementary Figure 5

Supplementary Figure 6

Supplementary Figure 7

Supplementary Figure 8

Supplementary Figure 9

Supplementary Figure 10
