# Supplementary material for: Transgenerational transmission of hedonic behaviors and metabolic phenotypes induced by maternal overnutrition
Source: Transl Psychiatry. 2018 Oct 12;8:195. doi: 10.1038/s41398-018-0243-2 (PMC6185972; doi:10.1038/s41398-018-0243-2)
Supplement: Supplementary file 1 — Supplementary File [file 41398_2018_243_MOESM1_ESM.pdf]

SUPPLEMENTARY INFORMATION

Transgenerational transmission of hedonic behaviors and metabolic phenotypes induced by maternal overnutrition.

Authors

[1]Gitalee Sarker, [2]Rebecca Berrens, [1]Judith von Arx, [3]Pawel Pelczar, [2]Wolf Reik, [1]Christian Wolfrum, [1*]Daria Peleg-Raibstein

Affiliations

[1]Laboratory of Translational Nutrition Biology, Department of Health Sciences and Technology, ETH Zurich, Schwerzenbach 8603, Switzerland.

[2]The Babraham Institute, Babraham, Cambridge CB223AT, United Kingdom.

[3]Center for Transgenic Models, University of Basel, Basel, Switzerland.

*Correspondence to: daria-peleg@ethz.ch

SUPPLEMENTARY MATERIALS AND METHODS

Experimental Design

The sample size selection, breeding and the experiments were designed according to the ARRIVE guidelines [1]. Random allocation of mice was conducted during breeding as well as during each experiment to exclude the selection bias. C57BL6/N mice obtained from Charles River, Germany were selected for breeding at the age of 10 weeks. To generate F1 offspring, 40 female mice were randomly divided into two groups (20 in each group) and were exposed to HFD or chow diet for 9 weeks (3 weeks preconception, 3 weeks gestation and 3 weeks lactation). 20 male breeders were randomly chosen and allocated as one male per 2 female breeders during the mating. To generate F2 and F3 offspring, 12 behaviorally naïve adult F1 males from HFD and CTR groups were randomly selected (one male per litter to exclude the litter effect) and mated with naïve primiparous females. All F2 and F3 animals were bred in identical breeding conditions. In each experiment, mice from multiple independent litters (one offspring per 10 different dams) were selected in each group to avoid the litter effect. Both male and female mice were chosen to assess the sex effects [2]. Behaviorally naïve mice were randomly allocated for each experiment (hedonic, metabolic, neuroanatomical and neurochemistry). Initially, the body weight of all offspring (F2 and F3) were measured since weaning to adulthood (PND 91). In both F2 and F3 generations, HFD preference test, sucrose preference test and alcohol preference test were conducted chronologically with 20 HFD (10 male and 10 female) and 20 CTR (10 male and 10 female) offspring. For the amphetamine sensitivity test, a separate group of mice (12 in each treatment group; 6 male and 6 female per group) was selected in both generations. Similarly, CPP test was performed in a different group of F2 and F3 offspring (24 mice in each HFD and control group). To assess the metabolic

parameters, a separate group of mice in both generations was allocated and the experiments were conducted in following orders: CT scan, metabolic cage study, and insulin sensitivity test and plasma insulin and lipid profiles. The behavioral and metabolic experiments were not blindly conducted. All the behavioral experiments (except CPP) and the metabolic experiments were replicated two times in two independently generated F2 as well as F3 cohorts. For neuroanatomical, neurochemical analysis and methylation assays, samples were collected from behaviorally naïve mice. For neuroanatomical analysis, 32 F2 offspring (16 HFD and 16 CTR) and 36 (18 HFD and 18 CTR) F3 offspring were used. For postmortem HPLC, 40 F2 (20 mice per group) and 36 F3 (18 mice per group) offspring were chosen. To perform sperm methylation assay, 12 F1 (6 HFD and 6 CTR) and 12 F2 (6 HFD and 6 CTR) fathers were selected. These experiments were blindly performed since each sample was recognized by a number throughout the experiment up to the raw data analysis. The samples were allocated in to the respected groups in the final statistical analysis.

Fat composition assay

Body fat composition was measured using a CT scanner (La Theta LCT-100; Aloka Inc, Japan). The scanner measures the volumes of adipose tissue, bone, air and the rest using differences in X-ray density. The X-ray source was set at 50 kV with a constant 1mA current. A mouse holder with an outer diameter of 48 mm and inner diameter of 41 mm was used, resulting in pixel resolutions of 100 µm on 480 × 480 pixel images. A pitch size of 1 mm was used. Mice were sedated by 4-5% isoflurane and maintained under gas anesthesia (1.5-2.5% isoflurane) while scanning. Animals were placed in supine position in the holder. The scan speed was set to 4.5 s/ image. A whole body scan excluding the head and the tail was performed for 5 min. Recorded scans were analyzed using the software La Theta 2.10.

Metabolic cage study

Metabolic phenotyping was performed in the TSE Phenomaster cages (TSE Systems GmbH, Bad Homburg, Germany). The TSE Phenomaster cages comprised of an indirect calorimetry system connected to a gas sensing unit to measure O_2 consumption (VO_2) and CO_2 production (VCO_2). A multi-dimensional infrared beam system assessed the locomotor activity as the total number of beam breaks in X and Y axis. Mice were single housed for 2 days prior to the experiment for acclimatization to the new cage environment. Room temperature was set to 23 °C; the light/dark cycle in the room was 12:12 h (lights off at 07:00 and lights on at 19:00). Mice were separately placed in the TSE Phenomaster cages and had ad libitum access to food and water. Mice were monitored for a total of 3 days. Body weight of each animal was measured before and after the experiment. O_2 consumption and CO_2 production were calculated from the gas concentrations and the air flow through the individual cage measured in parallel. Heat production, locomotor activity and food intake were also monitored during this period. Data were analyzed with Phenomaster software.

Amphetamine-induced locomotor activity

The locomotor response to a systemic amphetamine administration was tested in an open field apparatus. First, the mice were allowed to habituate to the open-field arena for 30 min (baseline). Then, they were administered an i.p. injection of saline (0.9% NaCl solution) and locomotor activity was measured for 30 min. The mice were then administered 2.5 mg/kg (i.p.) d-amphetamine sulphate (AMPH, Sigma-Aldrich, Switzerland) dissolved in 0.9% NaCl saline. The mice were placed back in the open field arena and the locomotor response to the acute drug challenge was measured for an additional 120 min. The locomotor activity was indexed by the distance travelled in the entire arena, expressed as a function of 12 successive

10 min bins. All solutions for injection were prepared on the day of experiment and were administered in a volume of 5 ml/kg body weight.

Conditioned place preference (CPP)

The CPP chamber consisted of two equally sized compartments with different colored walls and distinct floor textures that were separated by a small central area. A digital camera was directly mounted above the CPP apparatus which captured images at a rate of 5 Hz and transmitted them to the Ethovision tracking system (Noldus Technology). The distance travelled and total time spent in each compartment were recorded by the Ethovision software. The experiment consisted of three phases. During the pre-conditioning phase, all mice were allowed to freely explore all the compartments for 15 min. The conditioning phase lasted for eight days where the mice were conditioned to either cocaine or saline. During the conditioning phase, half of the animals from each group received the drug or vehicle in one compartment while the other half received it in the other compartment. During each of the four saline conditioning days (Day 1, 3, 5 and 7), an intraperitoneal (i.p.) injection of saline (0.9% NaCl) was administered to the mice immediately prior to confinement to the vehicle paired compartment for 30 min. On each of the four cocaine conditioning days (Day 2,4,6 and 8), mice received an i.p. injection of cocaine hydrochloride (Sigma–Aldrich, Switzerland) at a dose of 20 mg/kg before confining the mice to the drug-paired chamber for 30 min. The central area was made inaccessible by doors during conditioning. On the third phase, the preference test, took place 48 hours after the last conditioning day, the mice were again given free access to the entire apparatus without any injection for 30 min. The total time spent in each compartment was recorded during this period.

Dissection of brain regions

Adult mice from both groups were euthanized, the brains were rapidly removed, and 1 mm thick coronal sections were made. The slices were placed on an ice-chilled dissection plate for the extraction of the brain regions using a 1 mm micropunch. All brain areas of interest were dissected according to the Mouse Brain Atlas in Stereotaxic Coordinates (Paxinos and Franklin, 2008) which were as follows: dSTR (bregma +1.34 to +0.14 mm), Nac (bregma +1.60 to +0.98 mm), mPFC (bregma +2.68 to +1.94mm), Hypo (bregma -1.34 to -2.06mm), SN (bregma −2.80 to −3.64 mm), VTA (bregma −2.92 to −3.64 mm), Amyg (bregma from -0.94 mm to-2.30 mm), V. hip (bregma -2.54 to -3.80 mm) and D. hip (bregma -1.22 to -2.46 mm) and immediately frozen at −80°C.

Gene expression analysis of deltaFosB

The expression of delta FosB in dSTR and Nac of HFD and CTR offspring following chronic cocaine exposure was assessed by quantitative real time PCR (q-RT PCR). Total RNA from brain tissue was extracted using the Trizol-chloroform method (Invitrogen, CA, USA) according to the manufacturer's instruction and treated with DNase (Biolabs INc, CA, USA) for 30 min at 37°C. Following RNA extraction, 1µg of total RNA was converted to cDNA using High Capacity cDNA Reverse Transcription Kit [Applied Biosystems (ABI), Foster City, CA)]. The *qPCR* reactions were performed using the SYBER Green master mix (ABI) in the presence of primer pairs specific for the gene encoding for ΔFOSB. Endogenous control m36B4 was used to normalize the expression of the selected genes. Real time PCR reactions were conducted as follows: after a pre-denaturation and polymerase-activation program (5min at 95◦C), forty cycles each consisting of 95◦C for 3 sec and 60◦C for 30 sec were followed by a melting curve program (60 to 95◦C with heating rate of 0.1◦C/sec). The relative expression of mRNA was calculated using the 2- ΔΔCt method [1]. All primers were purchased from Microsynth AG, Switzerland. The primers used were as follows: ΔFOSB (forward, 5`-

AGGCAGAGCTGGAGTCGGAGAT-3`; reverse, 5`-GCCGAGGACTTGAACTTCACTCG-3`) and m36B4 (forward, 5'-GCCGTGATGCCCAGGGAAGA-3'; reverse, 5'-CATCTGCTTGGAGCCCACGTT-3').

Immunohistochemistry

Adult behaviorally naïve mice were deeply anaesthetized and perfused transcardially with phosphate buffer solution (PBS), followed by fixation with 4% phosphate-buffered paraformaldehyde solution containing 5% sucrose. After overnight fixation in the same fixative, the brains were cryoprotected using 30% sucrose solution and stored at 4°C. Later, the brains completely embedded in the cryomatrix (O.C.T; Thermo Scientific) were cut coronally at 40 µm thickness. The slices were first rinsed three times in PBS and then incubated with 0.5% H2O2 for 30 min. Later, the slices were incubated with 50mM ammonium chloride and subsequently with 50mM glycine buffer, for 30 min each. The sections were incubated overnight at 4◦C in primary antibody diluted in 0.5M Tris buffer, pH 8.0 containing 0.3% Triton X-100 and 4% normal goat serum. The following primary antibodies were used: rabbit anti-tyrosine hydroxylase (TH) (Santa Cruz Biotechnology sc14007; diluted 1:500), rat anti dopamine transporter (DAT) (Chemicon MAB369; diluted 1:1000), rat anti-dopamine D1 receptor (D1R) (Sigma Aldrich D2944; diluted 1:1000) and rabbit anti-dopamine D2 receptor (D2R) (Chemicon AB5084P; diluted 1:500). After overnight incubation with primary antibody and three washing steps in 0.1 M PB, the sections were incubated at room temperature for 1 h with the biotinylated secondary antibodies (Jackson Immunoresearch: diluted 1:500). The slices were then incubated in avidin-biotin-peroxidase complex (Vectastain PK6100 standard kit; Vector Laboratories) for 30 min. After three washing steps in 0.1 M PB, the sections were incubated for 5 min with 0.05% 3,3-diaminobenzidine (DAB), 0.03% Nickel ammonium sulfate and 0.02% H2O2 diluted in 0.05M Tris buffer (pH 8.0). After immunotaining, the sections were dehydrated, and cover slipped with DPX mountant.

Densitometry

Microscopic analysis was conducted under strictly blind conditions. Images were acquired at a magnification of 2.5× (NA 0.075) using a digital camera (Axiocam MRc5; Zeiss, Jena, Germany) attached to a Zeiss Axioplan microscope. Images were loaded into Image J software (ImageJ, NIH, Maryland, USA) for analysis. The pixel brightness was measured in non-immunoreactive areas of the forceps minor of the corpus callosum (for prefrontal cortical measures), corpus callosum (for dorsal striatal measurements) and anterior commissure (for nucleus accumbens' measures) as background area. The background-corrected relative optical densities were measured per brain region. Quantification was performed in four to six coronal brain sections and then averaged per brain area and animal.

Steriological analysis

The number of TH-positive neurons was determined by means of unbiased stereological estimations using the optical fractionator method. With the aid of the image analysis software Stereo Investigator (version 6.50.1; MicroBrightField), every section of a one-in-eight series was measured, resulting in an average of 5-6 sections per brain sample. The sampling parameters used were as follows: VTA (counting frame, grid size, sampling site=50um×50um, 120×130, 10; respectively) and SN (counting frame, grid size, sampling site=50um×50um, 150×110, 20; respectively). The counting frames were placed randomly at the intersections of the grid within the outlined structure of interest by the software. The cells were counted at the magnification of 20× [numerical aperture (NA), 0.40] according to the unbiased sampling rules and taken into the measurement when they came into focus within the optical dissector.

Post mortem HPLC

The brain regions isolated from adult mice were suspended in 0.4 M perchloric acid, sonicated and centrifuged at 10,000×g for 20 min at 4 ˚C. The supernatant was then collected, filtered and stored at -80 ˚C. The levels of dopamine (DA) and its metabolites DOPAC and HVA were measured by a high performance liquid chromatography (HPLC) system as previously described [3]. The sample (20 µl from each brain region) was injected by a refrigerated auto injector (ASI-100, Dionex, CA, USA). The HPLC mobile phase contained: 250 ml HPLC- grade acetonitrile, 5l aqueous solution having 0.27mM sodium ethylene-di-ammonium-tetra acetate ($C_{10}H_{14}N_2O_8Na_2•2H_2O$), 0.43mM triethylamine ($C_6H_{15}N$), 8 mM potassium chloride, and 0.925 mM octanesulphonic acid ($C_8H_{17}O_3SNa$), at pH 2.95. The mobile phase was passed through the system using a degasser at a flow rate of 0.4ml/min. Neurotransmitters were separated on a reversed-phase column (125mm×3mm YMC column, Nucleosil 120-3 C 18, YMC Europe GmbH, Germany) and the concentrations were detected using the electrochemical detector at an electrode potential of +0.70V. Data acquisition and calculations were performed using a chromatography workstation (Chromeleon, Dionex, Olten, Switzerland). Sample analysis was performed based on the position and height of the peaks of the endogenous components in relation to the samples of calibrating standard solutions.

Bisulfite sequencing analysis

Raw sequence reads were trimmed to remove both poor quality calls and adapters using Trim Galore (v0.4.1, www.bioinformatics.babraham.ac.uk/projects/trim_galore/, Cutadapt version 1.8.1, parameters: –paired). Trimmed reads were aligned to the mouse genome (GRCm38) in paired-end mode to be able to use overlapping parts of the reads only once. Alignments were carried out with Bismark v0.14.419 with the following set of parameters: paired-end mode: –pbat. Reads were then deduplicated with deduplicate_bismark selecting a random alignment

for position that was covered more than once. CpG methylation calls were extracted from the deduplicated mapping output, ignoring the first 6 bp of each read to reduce the methylation bias typically observed in PBAT libraries using the Bismark methylation extractor (v0.14.4) with the following parameters (i) paired-end mode: –ignore 6 –ignore_r2 6, (ii) single-end mode: –ignore 6. CpG methylation calls were analyzed using R and SeqMonk software (http://www.bioinformatics.babraham.ac.uk/projects/seqmonk/). 50 adjacent CpG running window probes were generated and percentage of methylation determined for probes containing at least five reads and three CpG on the pooled replicate data. The methylation level was expressed as the mean of individual CpG sites. Global CpG methylation levels of pooled replicates were illustrated using bean plots. Promoters were defined as the region -1 kb to the transcription start site as annotated in NCBIM37. For analysis of specific genome features these were defined as follows: Genic (overlapping coding sequences) were divided into exonic and intronic, CpG islands (CGI), promoters were defined as 1000 bp upstream of mRNAs, genic (overlapping coding sequences) were divided into exonic and intronic, repeat locations were extracted from the pre-masked RepeatMasker libraries (mm10 - Dec 2011 - RepeatMasker open-4.0.5, http://www.repeatmasker.org/species/musMus.html). Repeat instances overlapping annotated genes in the Ensembl gene set were removed to avoid mixing signals from genic expression with specific expression of repetitive sequences. Enhancers were taking from the super enhancers definied in ES cells. Graphing and statistics were performed using Seqmonk and RStudio.

In vitro fertilization

Spermatozoa extracted from HFD and CTR-F1 males were used to fertilize oocytes isolated from superovulated C57BL/6 females. The females at PND 28 were superovulated by i.p. injection of 5 IU of equine chorionic gonadotropin (PMSG) and 5 IU of human chorionic

gonadotropin (hCG) with the PMSG injection timed at 18:00 on day 1 and hCG at 18:00 on day 3, respectively. Next day, males were sacrificed, the dense sperm was removed from cauda epididymis and capacitated in 200ul of Fertiup medium (Cosmo Bio) for 45 minutes at 37°C. Following capacitation, 2ul of sperm solution was added to the IVF drop which comprised of 100 ul HTF medium (Cosmo Bio) overlaid with embryo tested mineral oil (Sigma). Females were then sacrificed, the oviducts were dissected immediately and the oocyte clutches were released directly into the IVF drop. The IVF reaction was conducted for 4 hours at 37°C. Following the IVF reaction, the efficiency of fertilization was confirmed by the presence of the pronuclei and the 2nd polar body. Fertilized oocytes were surgically transferred into pseudopregnant CD1 foster females previously mated with genetically vasectomized Prnm1GFP males and allowed to develop to term.

SUPPLEMENTARY FIGURE TITLES AND LEGENDS

Supplementary Figure 1. **Hedonic responses to natural rewards.**

(**a**) and (**b**) show the HFD preference in F2 and F3 generation respectively. The bar plots depict the consumption of HFD and chow diet per day normalized to body weight. (**c**) and (**d**) represent the sucrose preference in F2 and F3 offspring respectively. The bar plots show the mean sucrose consumption per day normalized to body weight in three different concentrations (0.5%, 1% and 3%). Data are represented as mean ± SEM. ***p< 0001. N (F2 CTR) = 20 (10 m, 10 f); N (F2 HFD) = 20 (10 m, 10 f); N (F3 CTR) = 20 (10 m, 10 f); N (F3 HFD) = 20 (10 m, 10 f); m = male, f = female.

Supplementary Figure 2. **Sensitization to cocaine in the F2 and F3 offspring.**

(**a**) and (**b**) represent the cocaine induced locomotor activity in F2 and F3 offspring

respectively. The line plots show the distance travelled (cm) in successive 5 min bins on the first day and following 21 days of cocaine withdrawal in response to a systemic injection of cocaine in the CPP paradigm. Data are represented as mean ± SEM. *$p<0.05$. N (F2 CTR) = 24 (12 m, 12 f); N (F2 HFD) = 24 (12 m, 12 f); N (F3 CTR) = 24 (12 m, 12 f); N (F3 HFD) = 24 (12 m, 12 f). m= male, f = female. (See also Figure 2).

Supplementary Figure 3. Neuroanatomical alterations in the dopaminergic system of the F2 and F3 offspring.

(**a-h**) show the representative images of coronal brain sections of F2 and F3 offspring for the expression of D1R in the dSTR and Nac. D1R = dopamine receptor 1, dSTR = dorsal striatum, Nac = nucleus accumbens, (See also Figure 3).

Supplementary Figure 4. Levels of dopamine (DA) and its metabolites (DOPAC and HVA) in post mortem brain tissue of the F2 offspring.

(**a**) shows the content of DA in the Hypo, mPFC, SN, Amyg, V. hip and D. hip in the F2 offspring. (**b**) and (**c**) display the levels of DOPAC in the dSTR, Nac, VTA as well as Hypo, mPFC, SN, Amyg, V. hip and D. hip respectively. (**d**) and (**e**) display the levels of HVA in the dSTR, Nac, VTA as well as Hypo, mPFC, SN, Amyg, V. hip and D. hip respectively. All monoamines contents are expressed as ng per mg fresh tissue weight. Data are represented as mean ± SEM. N (F2 CTR) = 20 (10 m, 10 f); N (F2 HFD) = 20 (10 m, 10 f). DA = dopamine, DOPAC = 3,4-Dihydroxyphenylacetic acid, HVA = homovanillic acid, dSTR = dorsal striatum, Nac = nucleus accumbens, VTA = ventral tegmental area, Hypo = hypothalamus, SN = substantia nigra, Amyg = amygdala, mPFC = medial prefrontal cortex, V. hip = ventral hippocampus and D. hip = dorsal hippocampus. m = male, f = female. (See also Figure 3).

Supplementary Figure 5. Levels of dopamine (DA) and its metabolites (DOPAC and HVA) in

post mortem brain tissue of the F3 offspring.

(**a**) shows the content of DA in the Hypo, mPFC, SN, Amyg, V. hip and D. hip in the F3 offspring. (**b**) and (**c**) display the levels of DOPAC in the dSTR, Nac, VTA as well as Hypo, mPFC, SN, Amyg, V. hip and D. hip respectively. (**d**) and (**e**) display the levels of HVA in the dSTR, Nac, VTA as well as Hypo, mPFC, SN and Amyg respectively. All monoamines contents are expressed as ng per mg fresh tissue weight. Data are represented as mean ± SEM. N (F3 CTR) = 18 (9 m, 9 f); N (F3 HFD) = 18 (9 m, 9 f). DA = dopamine, DOPAC = 3,4-Dihydroxyphenylacetic acid, HVA = homovanillic acid, dSTR = dorsal striatum, Nac = nucleus accumbens, VTA = ventral tegmental area, Hypo = hypothalamus, SN = substantia nigra, Amyg = amygdala, mPFC = medial prefrontal cortex, V. hip = ventral hippocampus and D. hip = dorsal hippocampus. m = male, f = female. (See also Figure 3).

Supplementary Figure 6. Altered metabolic phenotypes in the F2 and F3- HFD offspring.

(**a**) and (**b**) represent the total fat mass, subcutaneous and visceral fat depot in F2 and F3 offspring respectively. (**c**) and (**d**) display the area under the curve (AUC) for the insulin sensitivity test in the F2 and F3 offspring respectively. (**e**) and (**f**) show the concentration of triglycerides and free fatty acid (FFA) in the F2 and F3 offspring respectively. Data are represented as mean ± SEM. *$p<0.05$, **$p<0.01$. N (F2 CTR) = 12 (6 m, 6 f); N (F2 HFD) = 12 (6 m, 6 f); N (F3 CTR) = 12 (6 m, 6 f); N (F3 HFD) = 12 (6 m, 6 f). m = male, f = female. (See also Figure 4).

Supplementary Figure 7. Metabolic cage study in the F2 generation.

The bar plots represent average O_2 consumption (**a**), average CO_2 production (**b**), mean heat production (**c**), respiratory exchange ratio, RER (VCO_2/VO_2) (**d**) and physical activity (**e**) in 12 h dark and light cycle. The food intake per day was normalized to body weight (**f**). Data are

represented as mean ± SEM. *p<0.05, **p<0.01. N (F2 CTR) = 12 (6 m, 6 f); N (F2 HFD) = 12 (6 m, 6 f). m = male, f = female. (See also Figure 4).

Supplementary Figure 8. Metabolic cage study in the F3 generation.

The bar plots depict average O_2 consumption (**a**), average CO_2 production (**b**), mean heat production (**c**), respiratory exchange ratio, RER (VCO_2/VO_2) (**d**) and physical activity (**e**) in 12 h dark and light cycle. The food intake per day was normalized to body weight (**f**). Data are represented as mean ± SEM. *p<0.05. N (F3 CTR) = 12 (6 m, 6 f); N (F3 HFD) = 12 (6 m, 6 f). m = male, f = female. (See also Figure 4).

Supplementary Figure 9. Amphetamine induced locomotor activity in the IVF offspring.

The line plots show the distance travelled (cm) in successive 10 min bins following baseline, saline and a systemic injection of amphetamine in the open field. Data are represented as mean ± SEM. *p<0.05. N (F2 CTR) = 16 (8 m, 8 f); N (F2 HFD) = 16 (8 m, 8 f); N (F3 CTR) = 16 (8 m, 8 f); N (F3 HFD) = 16 (8 m, 8 f). m = male, f = female.

Supplementary Figure 10. Spem CpG methylation changes in F1 and F2 offspring.

(**a**) and (**b**) show the percentage of F2 DMRs found between F1 CTR and F1 HFD and F1 DMR found between F2 CTR and F2 HFD. The size of the dot represents the size of the DMR and the blue color scale indicates the p-value of the DMR.

REFERENCES

1. Kilkenny C, Browne WJ, Cuthill IC, Emerson M, Altman DG. Improving bioscience research reporting: the ARRIVE guidelines for reporting animal research. *PLoS biology* 2010; **8**(6)**:** e1000412.

2. Beery AK, Zucker I. Sex bias in neuroscience and biomedical research. *Neurosci Biobehav Rev* 2011; **35**(3)**:** 565-572.
