# Supplementary figures and images for: Transgenerational transmission of hedonic behaviors and metabolic phenotypes induced by maternal overnutrition
Source: Transl Psychiatry. 2018 Oct 12;8:195. doi: 10.1038/s41398-018-0243-2 (PMC6185972; doi:10.1038/s41398-018-0243-2)

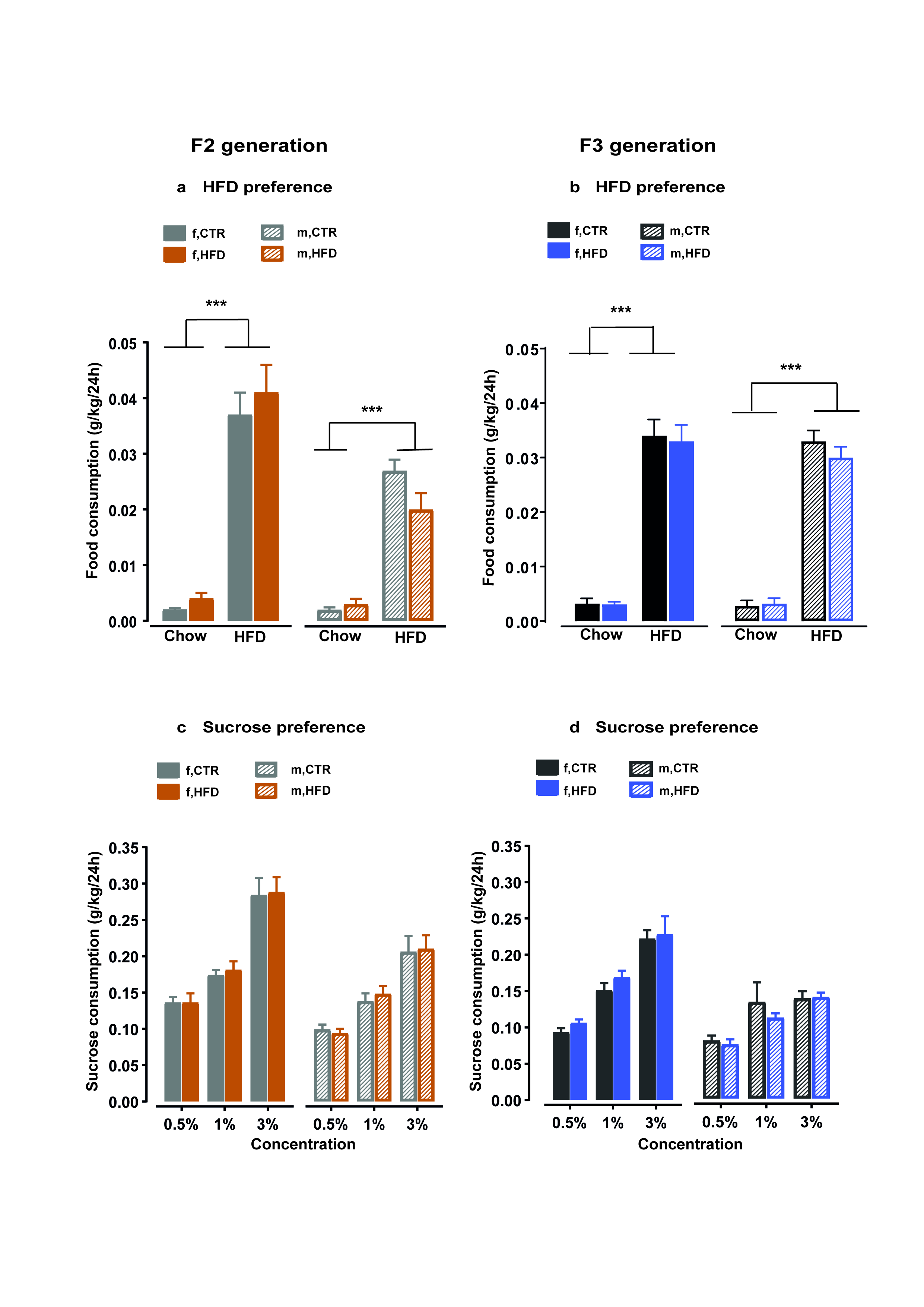

Supplement: Supplementary file 2 — Supplementary Figure 1 [file 41398_2018_243_MOESM2_ESM.tif]

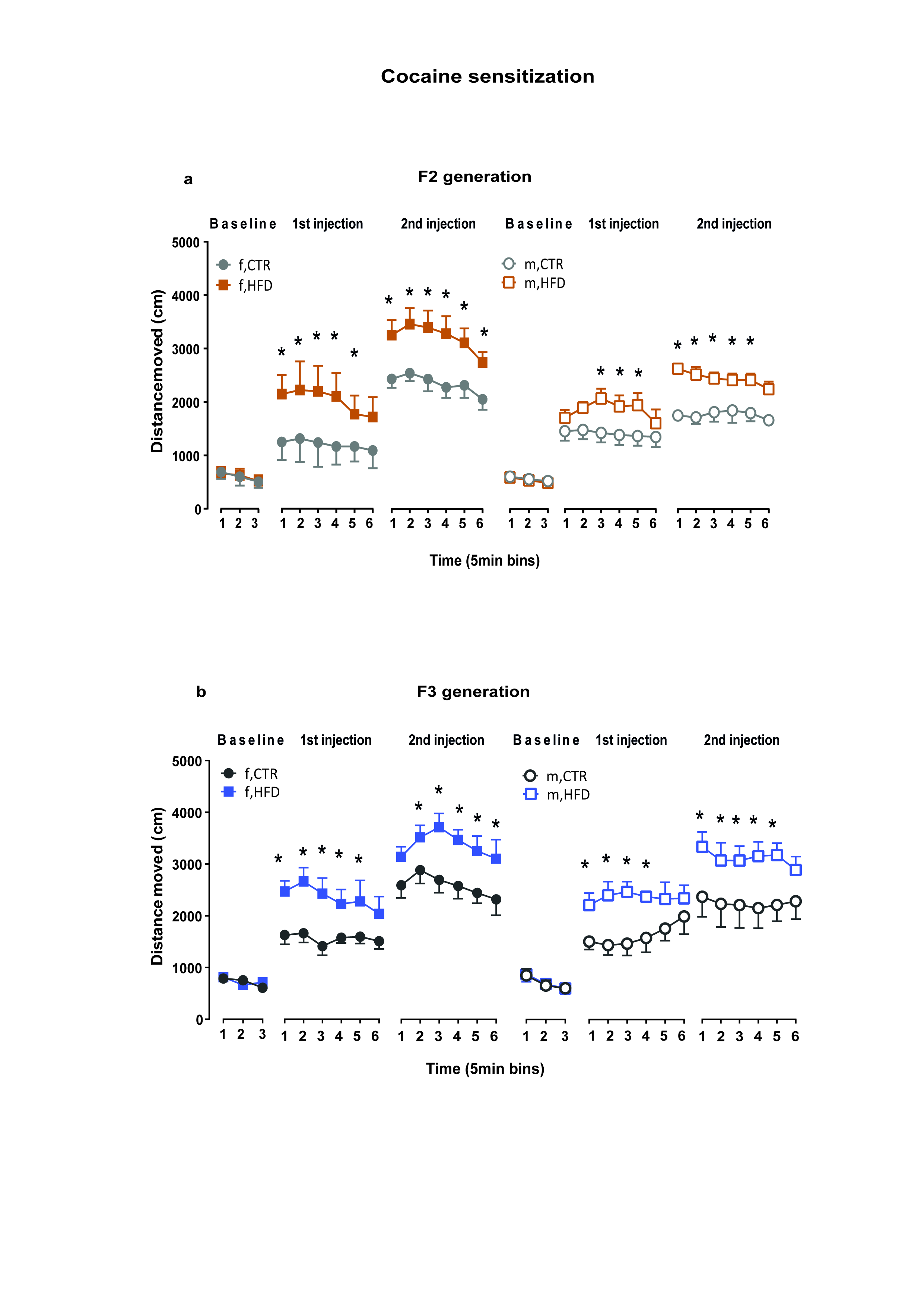

Supplement: Supplementary file 3 — Supplementary Figure 2 [file 41398_2018_243_MOESM3_ESM.tif]

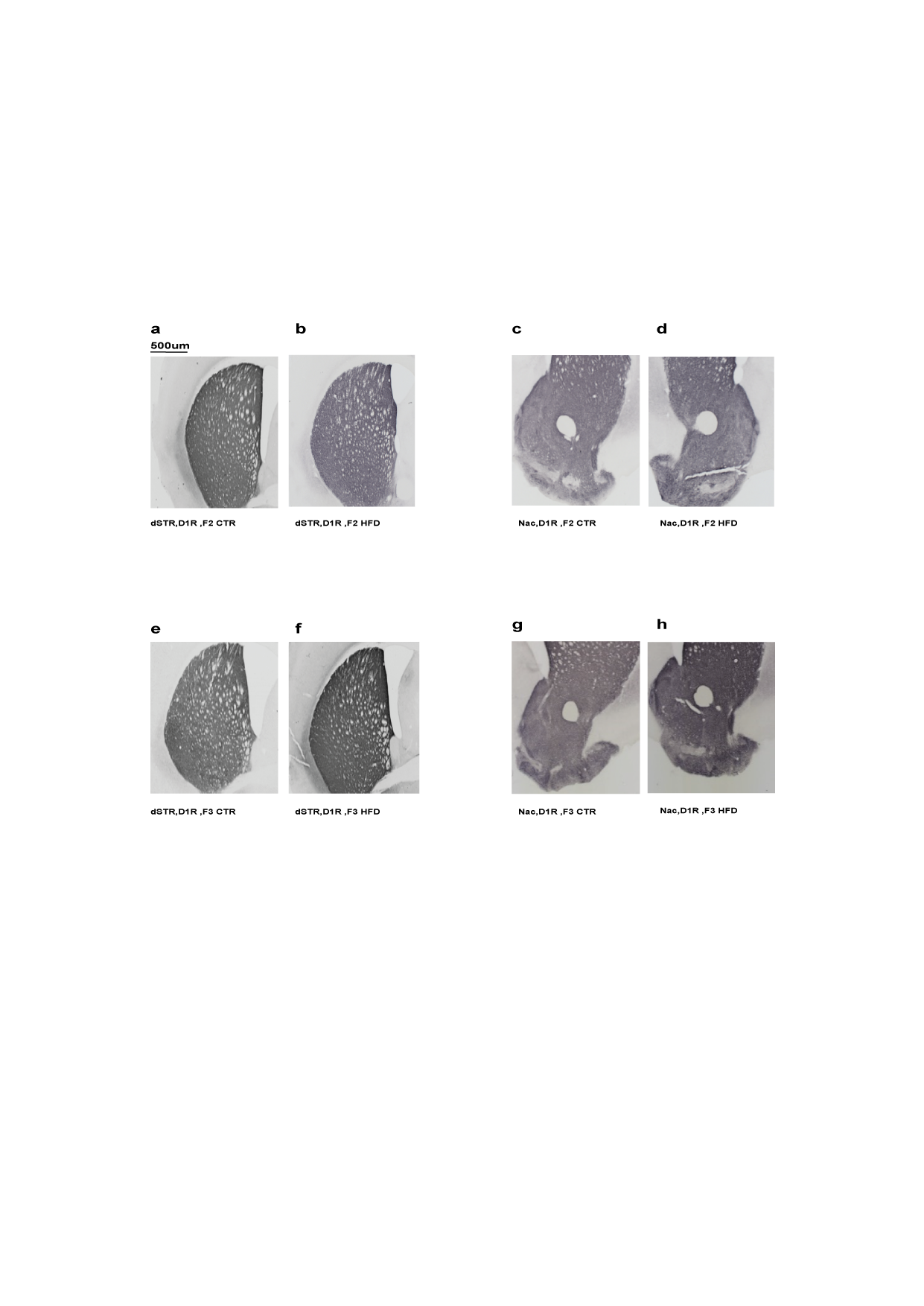

Supplement: Supplementary file 4 — Supplementary Figure 3 [file 41398_2018_243_MOESM4_ESM.tif]

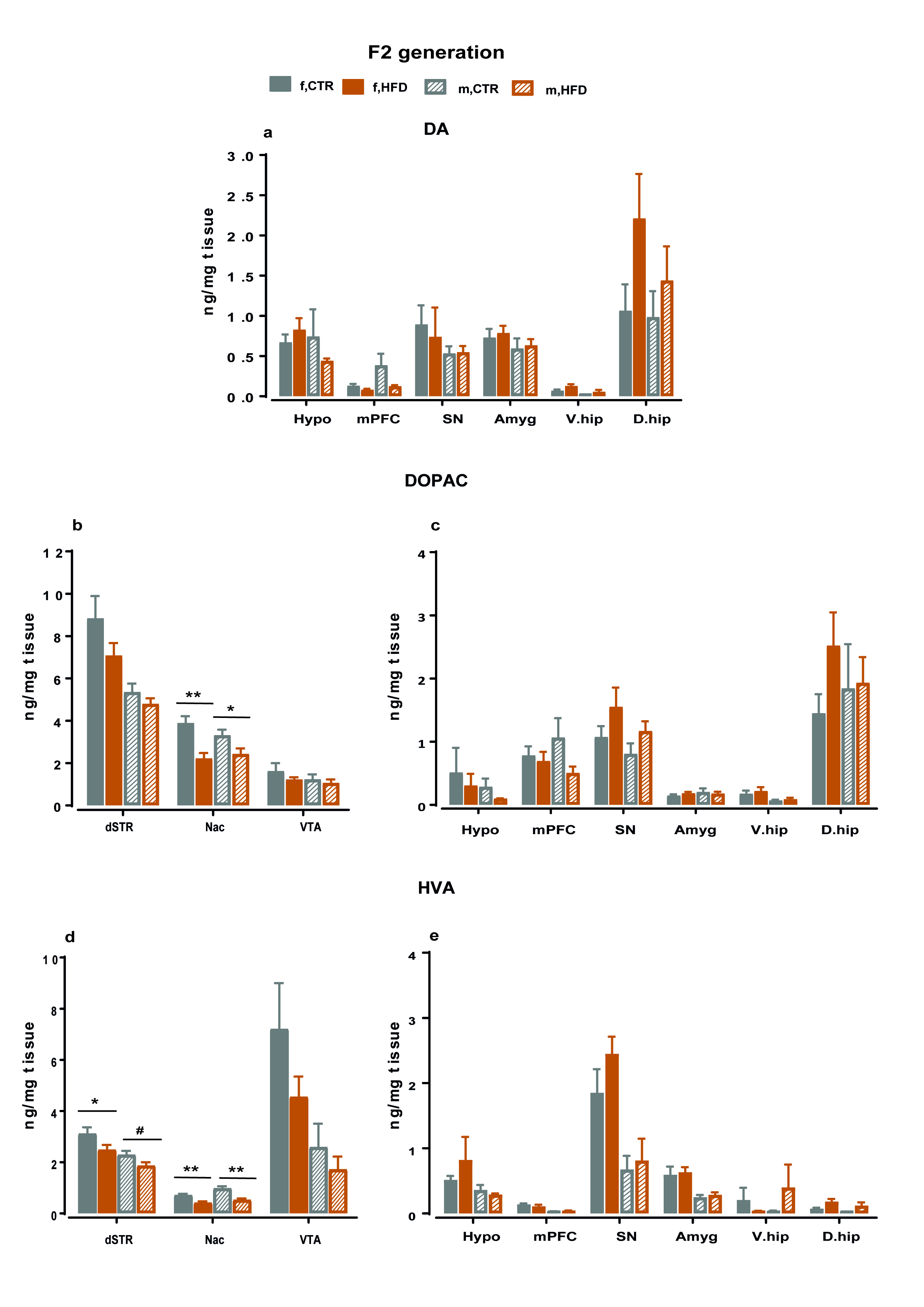

Supplement: Supplementary file 5 — Supplementary Figure 4 [file 41398_2018_243_MOESM5_ESM.tif]

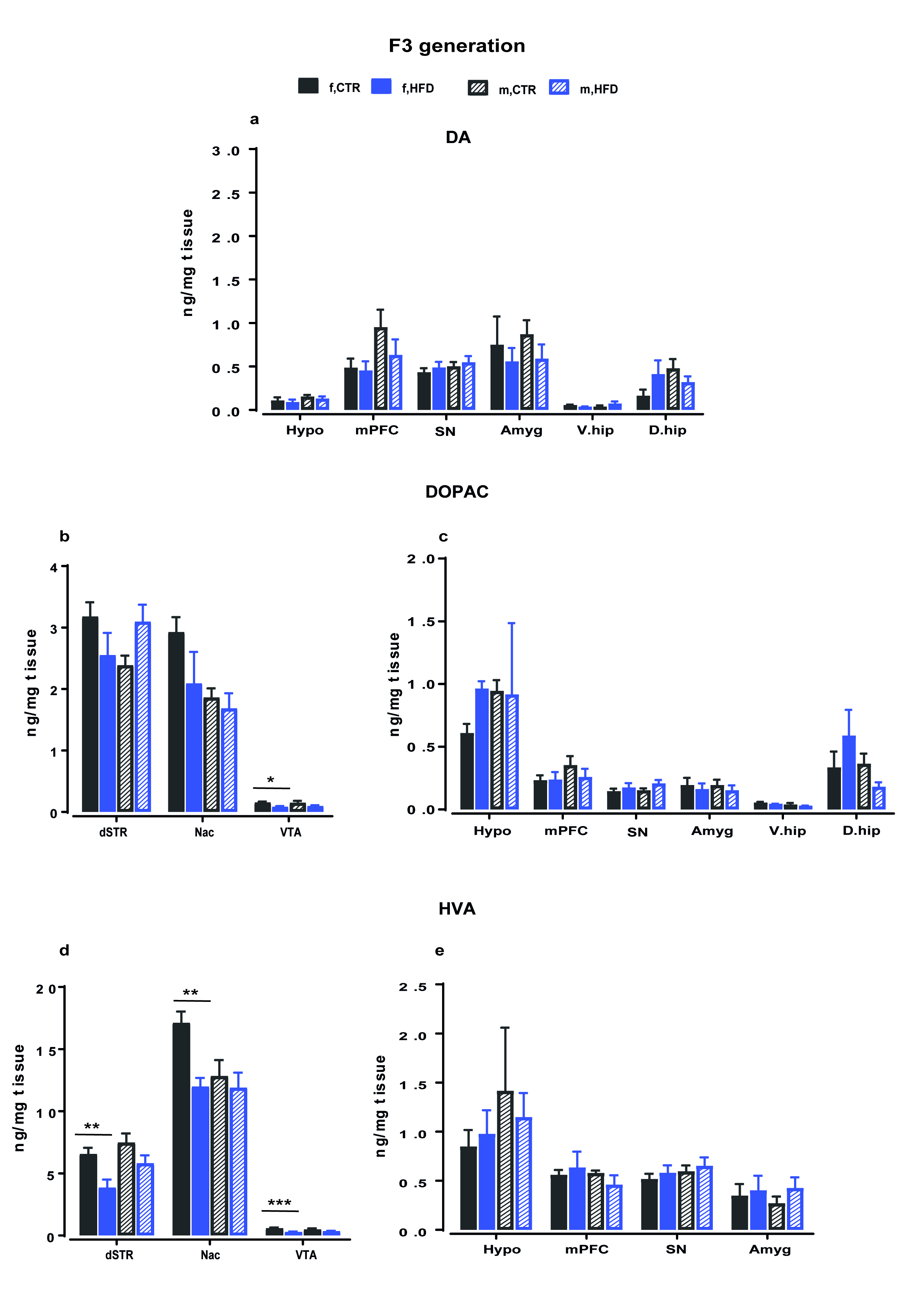

Supplement: Supplementary file 6 — Supplementary Figure 5 [file 41398_2018_243_MOESM6_ESM.tif]

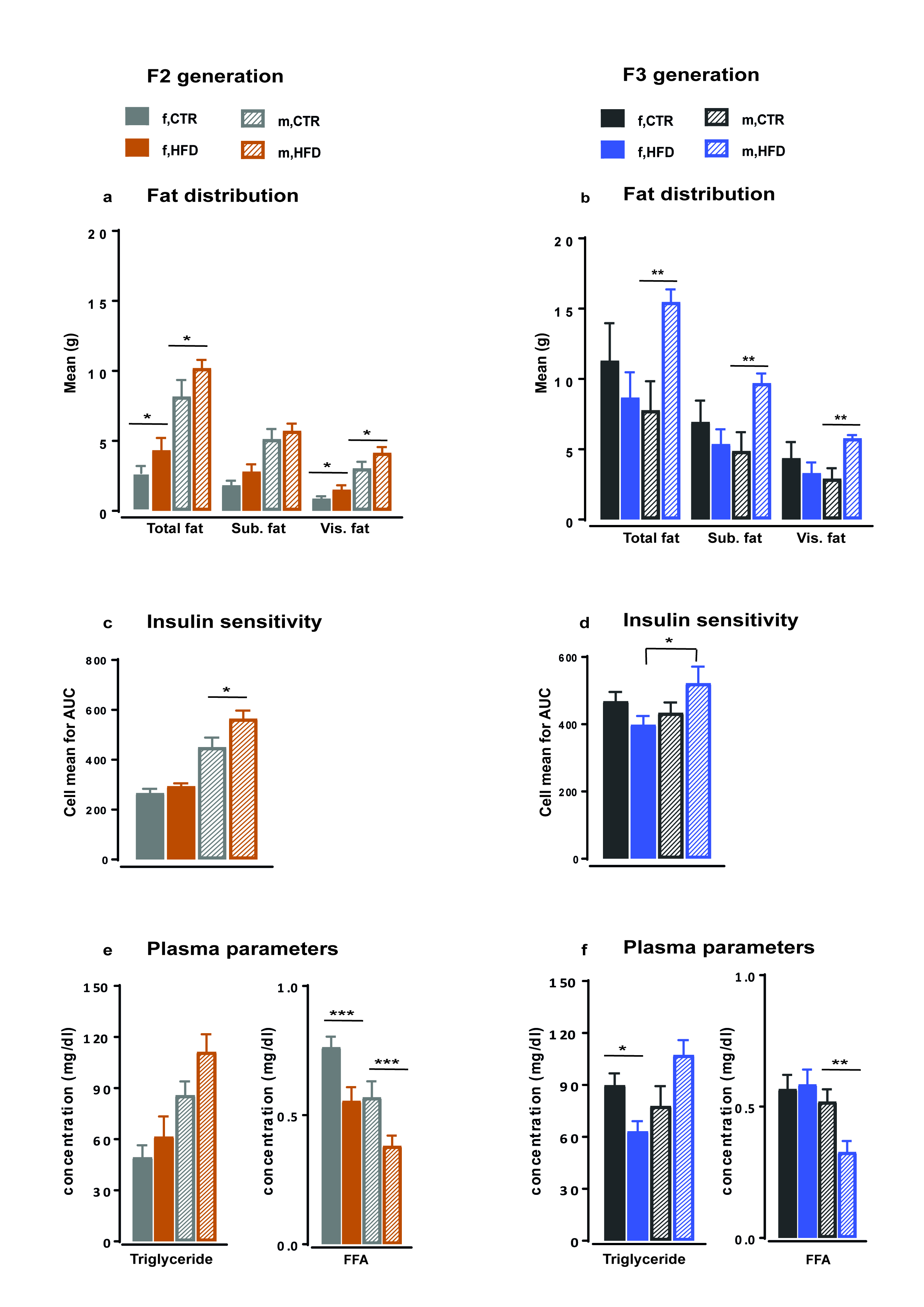

Supplement: Supplementary file 7 — Supplementary Figure 6 [file 41398_2018_243_MOESM7_ESM.tif]

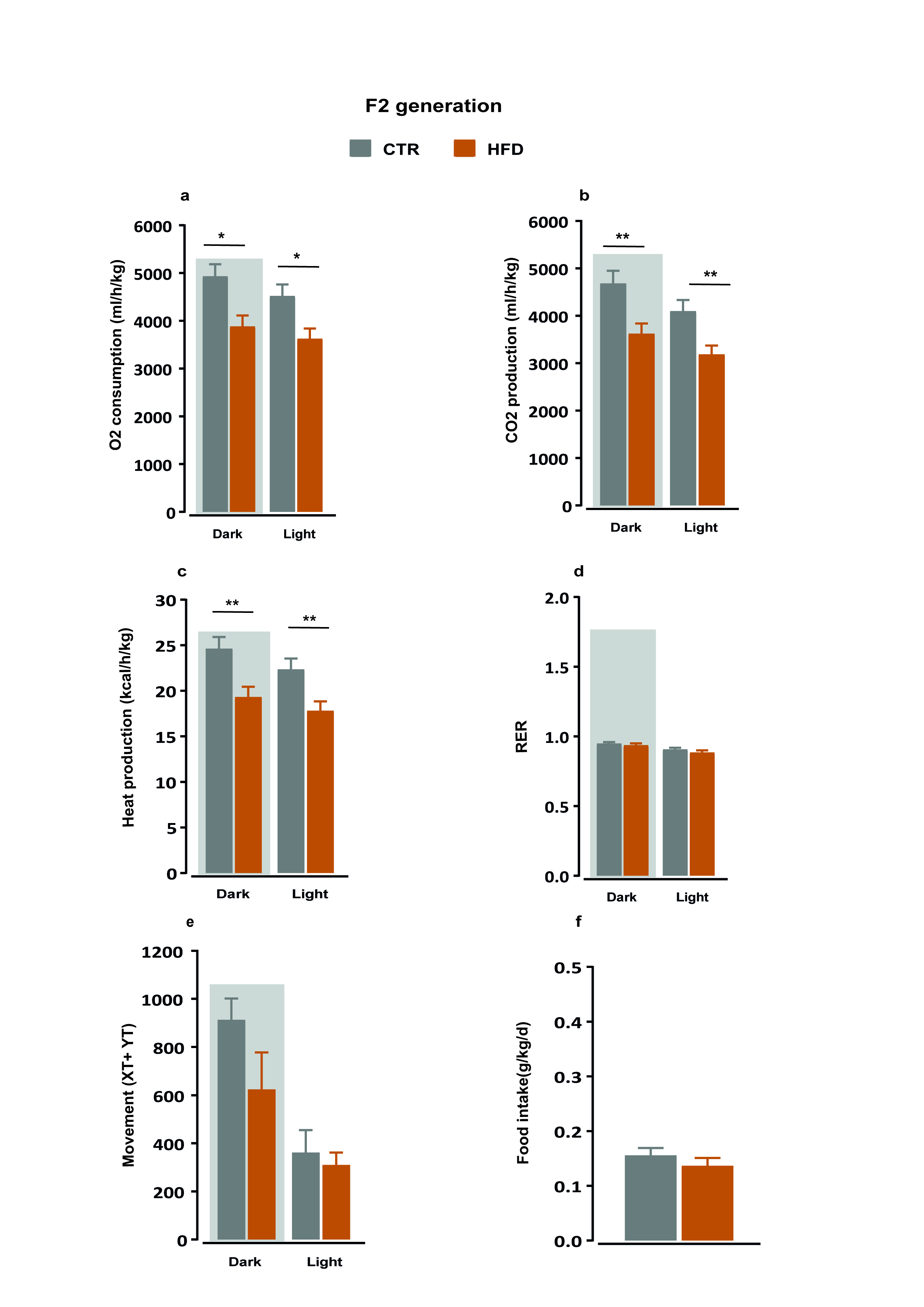

Supplement: Supplementary file 8 — Supplementary Figure 7 [file 41398_2018_243_MOESM8_ESM.tif]

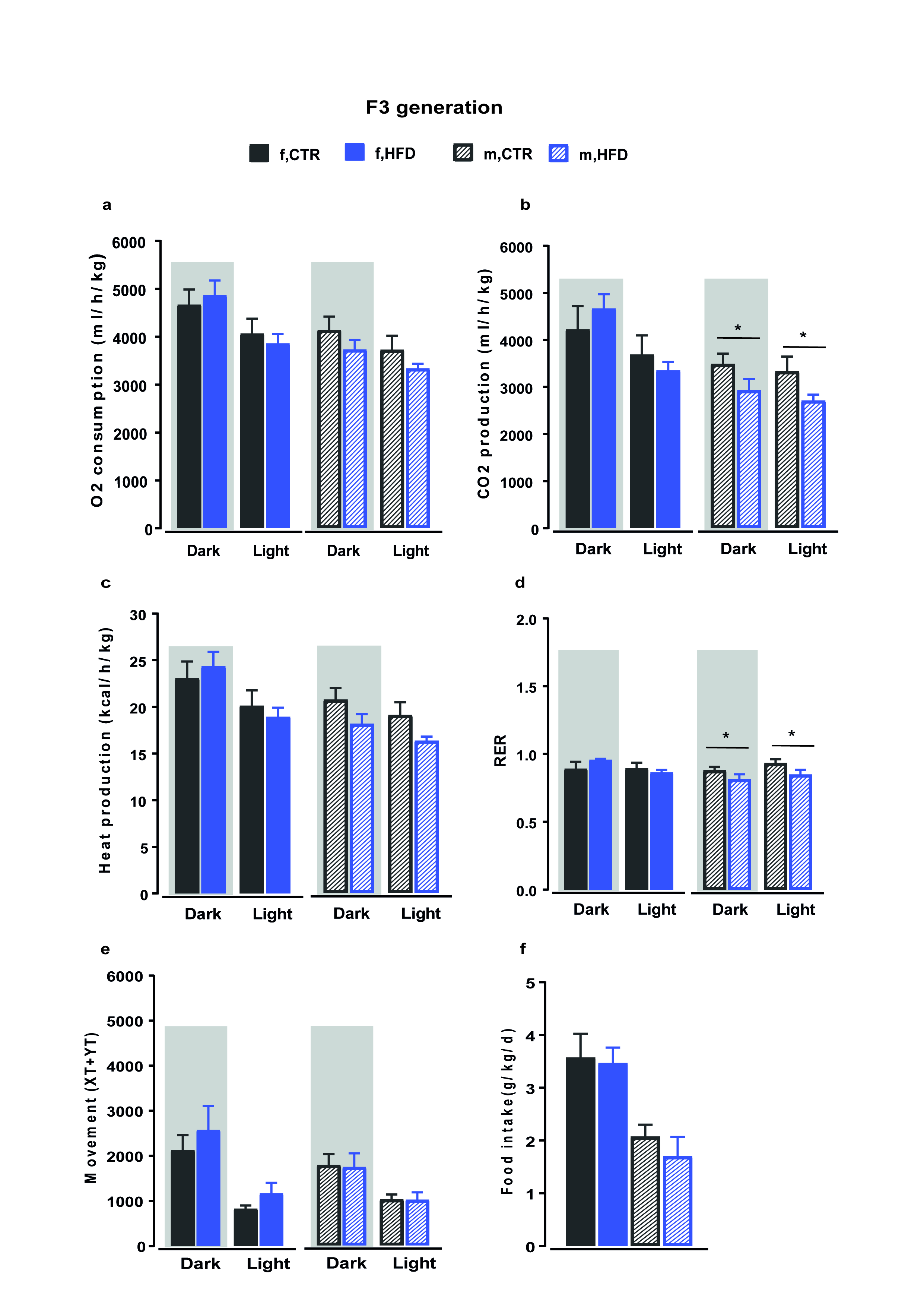

Supplement: Supplementary file 9 — Supplementary Figure 8 [file 41398_2018_243_MOESM9_ESM.tif]

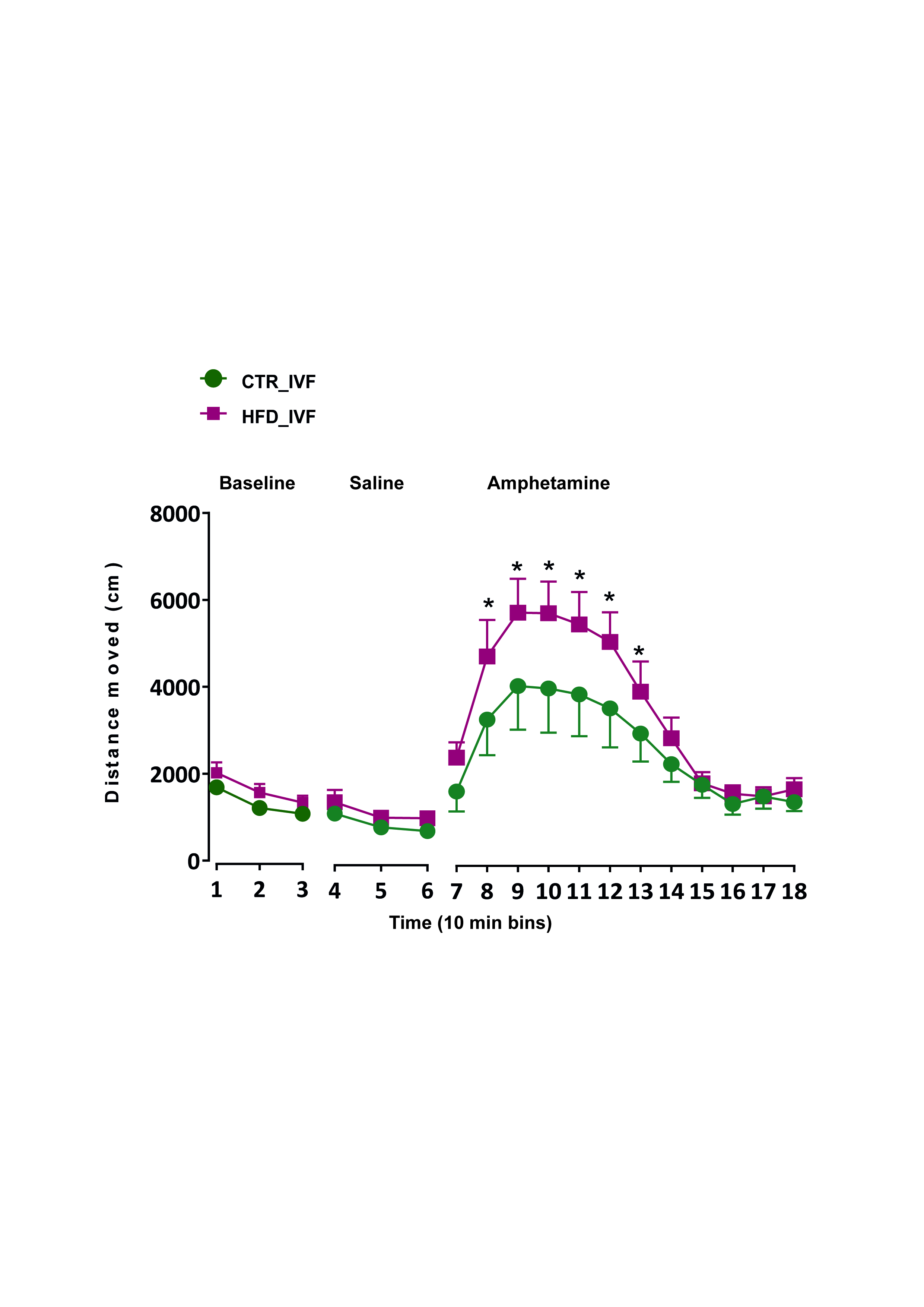

Supplement: Supplementary file 10 — Supplementary Figure 9 [file 41398_2018_243_MOESM10_ESM.tif]

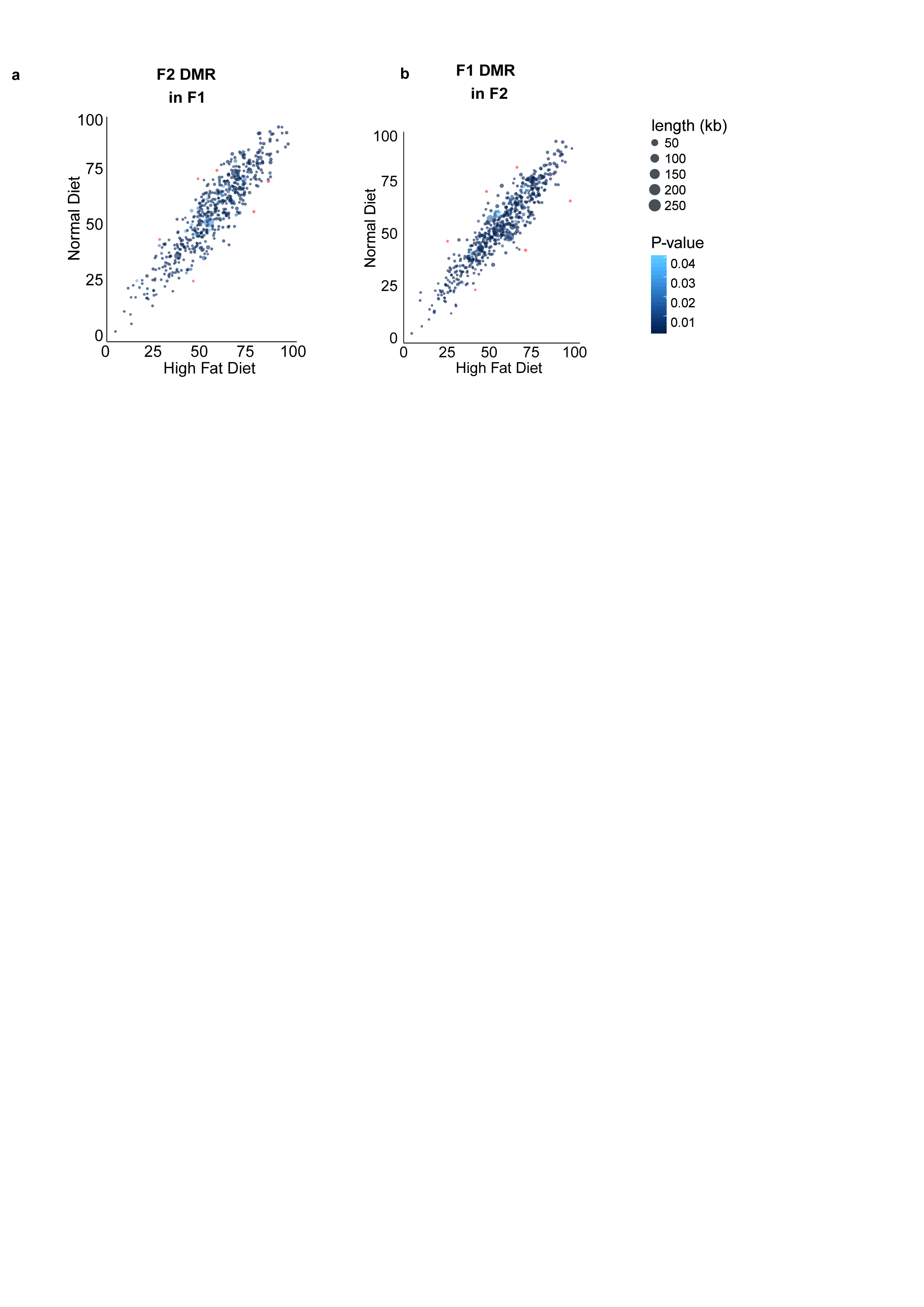

Supplement: Supplementary file 11 — Supplementary Figure 10 [file 41398_2018_243_MOESM11_ESM.tif]
